# Molecular Mechanisms of Plant Responses to Copper: From Deficiency to Excess

**DOI:** 10.3390/ijms25136993

**Published:** 2024-06-26

**Authors:** Ending Xu, Yuanyuan Liu, Dongfang Gu, Xinchun Zhan, Jiyu Li, Kunneng Zhou, Peijiang Zhang, Yu Zou

**Affiliations:** 1 Anhui Province Key Laboratory of Rice Germplasm Innovation and Molecular Improvement, Rice Research Institute, Anhui Academy of Agricultural Sciences, Hefei 230031, China; 2 Department of Biochemistry & Molecular Biology, College of Life Science, Nanjing Agriculture University, Nanjing 210095, China; 3 Institute of Horticultural Research, Anhui Academy of Agricultural Sciences, Hefei 230031, China

**Keywords:** copper, deficiency and phytotoxicity, transporter, transcriptional regulation, homeostasis

## Abstract

Copper (Cu) is an essential nutrient for plant growth and development. This metal serves as a constituent element or enzyme cofactor that participates in many biochemical pathways and plays a key role in photosynthesis, respiration, ethylene sensing, and antioxidant systems. The physiological significance of Cu uptake and compartmentalization in plants has been underestimated, despite the importance of Cu in cellular metabolic processes. As a micronutrient, Cu has low cellular requirements in plants. However, its bioavailability may be significantly reduced in alkaline or organic matter-rich soils. Cu deficiency is a severe and widespread nutritional disorder that affects plants. In contrast, excessive levels of available Cu in soil can inhibit plant photosynthesis and induce cellular oxidative stress. This can affect plant productivity and potentially pose serious health risks to humans via bioaccumulation in the food chain. Plants have evolved mechanisms to strictly regulate Cu uptake, transport, and cellular homeostasis during long-term environmental adaptation. This review provides a comprehensive overview of the diverse functions of Cu chelators, chaperones, and transporters involved in Cu homeostasis and their regulatory mechanisms in plant responses to varying Cu availability conditions. Finally, we identified that future research needs to enhance our understanding of the mechanisms regulating Cu deficiency or stress in plants. This will pave the way for improving the Cu utilization efficiency and/or Cu tolerance of crops grown in alkaline or Cu-contaminated soils.

## 1. Introduction

Copper (Cu) is an essential element for living organisms and plays an important role in numerous biological processes. In humans, Cu acts as a cofactor for a variety of enzymes, such as Cu/zinc (Zn) superoxide dismutase (Cu/Zn-SOD, CSD), cytochrome c oxidase, mitogen-activated protein kinase MEK1, and CAMP-degrading phosphodiesterase PDE3B. Insufficient Cu intake in humans can lead to Cu deficiency disorders such as anemia, pancytopenia, and ataxia [1]. Chronic Cu poisoning occurs when humans are exposed to excessive amounts of Cu for a long period of time. Cu toxicity often presents with the development of cirrhosis, accompanied by hemolytic attacks and damage to the renal tubules, brain, and other organs [2]. Cu is one of the 17 essential elements required for plant growth. Plants have low requirements for this element; however, similar to other essential nutrients, it is crucial for their growth. Under physiological conditions, Cu participates in various cellular metabolic processes [3]. Despite its importance in metabolic activity, little research has been conducted on its role in plant homeostasis. Although Cu is abundant in most soils, specific soil pH and redox conditions significantly influence the concentration of Cu. Cu deficiency in plants typically occurs in calcareous alkaline soils with a high pH or soils with a high organic content, which can result in severe nutritional disorders [4,5]. Although Cu deficiency can be addressed using Cu-based fertilizers, this approach is not environmentally friendly and may result in the accumulation of toxic Cu in the soil [6]. Cu availability is generally higher in acidic soils, which increases its potential toxicity to plants [7]. Owing to prolonged industrial activities and the widespread use of Cu-containing fungicides in agricultural production, a significant amount of Cu is released into the soil. This influx of Cu may be a key factor contributing to Cu toxicity in plants [8]. It has been reported that the application of certain soil amendments can alter the soil pH, thereby influencing the bioavailability of Cu in the soil [9]. The application of lime can promote the alkalization of acidic soil solutions, thus inducing the deprotonation of acid functional groups on the surface of soil particles, increasing the cation exchange capacity of the soil, and enhancing the adsorption of Cu [10]. However, this is a temporary solution. Owing to the potential leaching of Cu along soil profiles, the increased mobility of Cu may pose an environmental risk. Some researchers have applied fly ash to Cu-polluted soil to increase the soil pH and reduce the uptake of Cu by plants [11]. However, this strategy is costly and often impractical for farmers with poor-quality land.

In response to changes in external Cu levels, plants must either efficiently acquire and use Cu under limited conditions or detoxify the metal with excess supply. Plants have evolved complex molecular regulatory mechanisms to maintain Cu homeostasis and ensure normal life activities during long-term environmental adaptation [12]. To date, various Cu transporters, chaperones, and chelators have been identified to be involved in Cu uptake, transport, and maintenance of cellular homeostasis in plants. This review discusses the functions of Cu-transport-related proteins in various plant species. Table 1 lists the Cu transporters discussed in the article.

## 2. Cu Is an Essential Element in Plant Metabolism

Cu plays a crucial role in various processes of the plant life cycle, such as photosynthesis, respiration, ethylene sensing, clearance of reactive oxygen species (ROS), cell wall metabolism, and hormone signaling [48,49]. As a redox-active metal, Cu can cycle between the reducing state Cu^1+^ and the oxidizing state Cu^2+^ under physiologically relevant conditions, thereby catalyzing single-electron transfer reactions [50]. The reduced form of Cu^1+^ preferentially binds sulfur-containing compounds with mercaptan or thioether groups, whereas the oxidized form of Cu^2+^ mainly coordinates with the oxygen or imidazole nitrogen groups [51,52]. Therefore, Cu acts as a structural or catalytic cofactor in essential cellular enzymes that take advantage of its redox potential.

It is estimated that 30% of Cu is present in the chloroplasts of green plants [53]. However, 60–80% of Cu in chloroplasts is present in thylakoids, indicating the important role of Cu in photosynthetic proteins and complexes [53,54]. Plastocyanin (PC), a small soluble Cu protein in the thylakoid lumen, plays a crucial role in both linear and cyclic photosynthetic electron transport and is essential for the photoautotrophic growth of plants [55]. PC is one of the most abundant proteins in the thylakoid membrane. It was first isolated from Chlorella and has since been found in cyanobacteria, algae, and plant chloroplasts [55]. During photosynthesis, PC serves as an electron carrier from cytochrome *f* (Cyt *f*) in the cytochrome *b_6_f* complex (Cyt *b_6_f*) to P_700_ in photosystem I (PSI) [56,57]. Some studies have shown that Chlamydomonas can use heme-containing cytochrome *c_6_* (Cyt *c_6_*) to functionally replace PC when Cu is limited [58]. However, in terrestrial plants, the modified form Cyt *c_6A_* of Cyt *c_6_* is not suitable for use as an electron carrier between cytochrome b6f and PSI because of its unique topological structure; therefore, it cannot replace PC in Arabidopsis [59,60]. Two PC subtypes are encoded in most angiosperms, including Arabidopsis [61], poplar [62], tobacco [63], rice [64], and *Physcomitrella patens* [65]. These two subtypes have essentially the same function in terms of electron transport activity. However, in Arabidopsis, one protein subtype (PC1) accumulates at lower levels, whereas the second subtype (PC2) is more abundant but also more sensitive to Cu availability at the protein level [66,67]. Polyphenol oxidase (PPO), a Cu-rich enzyme, is found in the thylakoid cavity of many plants (such as poplar and spinach). It catalyzes the conversion of monophenol to o-dihydroxyphenyl and o-quinone, which easily leads to black or brown pigmentation. It is thought to play a role in defense against attacks by herbivores or pathogens [68,69,70].

Cu serves as a cofactor for the terminal enzyme complex cytochrome c oxidase (COX, or Complex IV) in the mitochondrial respiratory chain [71,72]. The cytochrome c reductase complex (Complex III) transfers electrons from panthenol to cytochrome c. Finally, COX accepts electrons from cytochrome c, facilitating the conversion of oxygen and H into water [73]. Eukaryotic COX has 11–13 subunits, with the three largest subunits (COX1, COX2, and COX3) encoded by the mitochondrial DNA [74,75,76]. The normal function of COX requires five types of cofactors: two hemes, three Cu ions and Zn, manganese (Mn), and sodium (Na) ions [76].

Multicopper oxidase (MCO) is a superfamily of proteins present in a wide variety of organisms that binds to four Cu ions [77,78]. In plants, MCO consists mainly of ascorbate oxidase and laccase [78]. Ascorbate oxidase is found in exosomes and plays an important role in cell expansion, salt tolerance, and plant productivity [79,80]. Laccase (LAC), similar to ascorbate oxidase, is present in exosomes and uses molecular oxygen as the final electron acceptor to catalyze the oxidation of various substrates (such as phenols, aromatics, or aliphatic amines) to generate reactive radicals. These radicals then undergo non-enzymatic oxidation or reduction reactions to produce water and oligomers [81]. LAC plays an important role in various physiological and biochemical processes, including lignin and flavonoid biosynthesis [82,83], pigmentation [84], maintenance of the cell wall structure and integrity [82], and salt stress tolerance [85]. Another Cu protein found in exosomes is plantacyanins, which belong to the phytocyanin family of blue Cu proteins and play a role in guiding pollen tubes [86].

CSD is a Cu protein and one of the three common types of superoxide dismutase (SOD) in plants: CSD, Mn-SOD, and Fe-SOD [87]. CSD can catalyze the conversion of superoxide free radicals into molecular oxygen and hydrogen peroxide, which play a crucial role in scavenging ROS and alleviating oxidative and abiotic stress [88]. Three subtypes of CSD have been identified in *Arabidopsis thaliana*: CSD1 in the cytoplasm, CSD2 in the chloroplast, and CSD3 in the peroxisome [89]. Evidence suggests that the Cu companion of superoxide dismutase (CCS) is a key factor in the integration of Cu into CSD in eukaryotes [90,91].

Ethylene signaling is another Cu-dependent process [92]. Cu is a cofactor for ethylene receptors. Ethylene affects many aspects of plant life, including seed germination, stem thickening, fruit ripening, stomatal opening, and plant responses to biotic and abiotic stresses [92,93,94]. Ethylene is sensed by a series of membrane-binding receptors that are negative regulatory molecules in the ethylene reaction. When the receptors bind to ethylene, their activation response signaling is turned off [95].

Cu is also involved in the synthesis of molybdenum cofactors [96], the biosynthesis of quinones and carotenoids [97,98], pollen formation [99], and oxidative phosphorylation [98]. Cu can also enhance the flower and vegetable yields, flavor, color, sugar content, and storage life of fruits [100,101,102]. Moreover, Cu ions are widely used in agriculture owing to their antibacterial, antiviral, and insect-resistant properties [103,104].

## 3. Cu Dynamics in Soil

**Figure 1:**
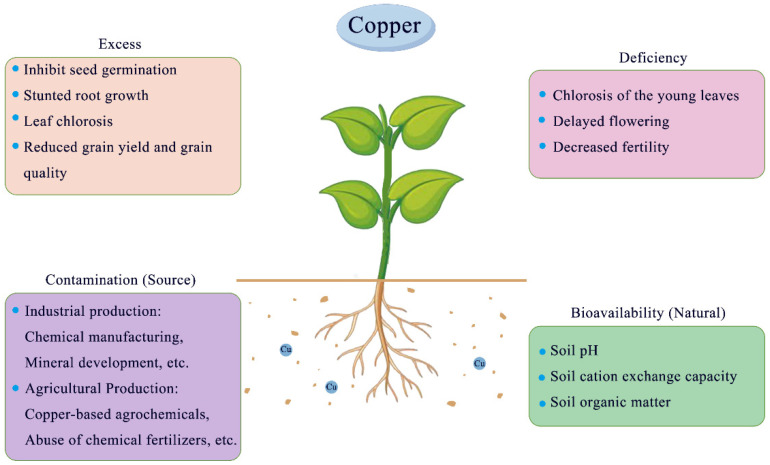
Overview of Cu sources, soil bioavailability, and the effects of its deficiency and excess on plant systems (for details, see text).

Cu is a reddish-brown transition metal with a natural abundance of 60 mg/kg in Earth’s crust [105]. The natural form of Cu usually exists in two forms: the reduced cuprous ion, Cu (I), and the Cu oxide ion, Cu (II) [106]. In soil solutions, Cu can exist in its free form, such as Cu^2+^. However, in most cases, Cu is complexed with inorganic anionic binders or organic molecules. Approximately 80% of Cu in soil exists in the form of insoluble compounds (oxides and sulfides), which are not conducive to plant utilization [9]. In contrast, approximately 20% of Cu in the soil is in the soluble form (hydroxyl and carbonate), which is typically available to plants. However, the bioavailability of soluble Cu depends on the functional groups of the active soil particles and their adsorption capacity, which are influenced by various factors [107] (Figure 1). It is generally believed that a soil Cu content <8 mg/kg may cause Cu deficiency symptoms in crops [108]. Varying degrees of Cu deficiency exist in agricultural soils in many areas of the world, adversely affecting the yields of rice, wheat, and corn [109,110,111,112,113].

The adsorption capacity of Cu increased with higher levels of clay minerals, iron (Fe), aluminum (Al), Mn hydroxide or oxygen hydroxides, carbonate, and organic matter [114,115]. Soil pH, cation exchange capacity, and soil organic matter (SOM) content are important factors in regulating the conversion between the solubility, adsorption, desorption, and form of heavy metals (including Cu) and, therefore, the availability of Cu [49]. It has been reported that the available Cu content in the soil is significantly positively correlated with soil pH [116]. When the soil pH decreases, the Cu adsorbed or co-precipitated by carbonate can easily be re-released into the environment. Additionally, some evidence suggests that plants can alter the pH of the soil around their roots, thereby altering the bioavailability of metals in the root environment [117,118]. For example, plant root exudates (e.g., citric acid and malic acid) can cause the rhizosphere pH to be more acidic than that of bulk soil, leading to the dissolution of Cu, which may then be absorbed near the roots [117,119]. In addition to soil pH, SOM content significantly influences the fluidity and bioavailability of heavy metals in the soil [120]. SOM is the main adsorbent that controls the distribution of heavy metals in soil solids and solutions. Its main components are humic and fulvic acid, which are organic compounds with complex structures [121]. These structures include alkyl, carbonyl, sugar residues, heteroatoms, and various other functional groups [121]. The complexation of heavy metals with organic ligands affects their mobility by either increasing or decreasing their adsorption onto the mineral surfaces [122,123]. Cu and SOM form strong complexes; therefore, Cu–SOM complexes are usually the dominant form present in soils with a high SOM content [124]. Owing to the high affinity of SOM for Cu, the effect of soil pH on the fluidity and availability of Cu diminishes significantly when SOM content is high [125]. Therefore, plants growing in swampy, humus, calcareous, or alkaline soils with high organic matter content in arid and semi-arid environments may develop Cu deficiencies.

In addition to the physical and chemical properties of the soil, industrial production and human activities are important factors affecting the bioavailability of Cu in the soil (Figure 1). Industrial production includes factories that manufacture or use Cu metal or related compounds, Cu mining and drainage, fertilizer production, and chemical manufacturing. These activities often lead to the release of high levels of Cu into the soil [126,127]. It is estimated that 939,000 tons of Cu have been discharged from the natural environment worldwide over the past 50 years [128]. It has been reported that the Cu content in Chinese soil ranges from 0.3 to 272 mg/kg, with an average of 21.9 mg/kg [129]. However, in some seriously polluted areas, such as near mining sites, the Cu content in the soil can reach levels as high as 5000 mg/kg. When the Cu content in the soil exceeds 50–250 mg/kg, it can negatively affect plant growth [129]. Furthermore, other anthropogenic activities associated with agricultural practices, especially the application of Cu-based agrochemicals such as fertilizers, pesticides, fungicides, and herbicides, are major sources of Cu deposition in soil [130]. For example, to prevent citrus canker, some citrus growers often use Cu-containing fungicides such as Bordeaux liquid, Cu hydroxide, Cu oxychloride, and thiazide Cu, as well as excessive application of Cu-containing trace element fertilizers. This practice leads to the annual accumulation of Cu on the surface of the orchard soil, consequently increasing Cu levels in both soil and trees [131,132]. Moreover, some fruit farmers unilaterally pursue economic benefits by reducing the input of organic fertilizer and applying a high content of acid compound fertilizer, along with undecomposed livestock and poultry manure, leading to soil acidification [133]. Therefore, excess available Cu is common in citrus orchard soils. Research has continued to develop effective remediation methods to treat contaminated land and mitigate the negative impacts of Cu infiltration into the ecosystem. However, the choice and practicality of remediation measures depend on various factors, such as availability, cost, and duration of effectiveness. For example, farmers can minimize the toxicity of Cu to plants by using soil amendments (such as adding biochar, lime, and phosphate) to reduce the availability of Cu in the soil [108]. However, the use of these soil amendments can increase farming costs, reduce herbicide effectiveness, and introduce other pollutants that have potentially negative effects on soil bacteria and animals.

## 4. Cu Deficiency and Toxicity

### 4.1. Cu Deficiency

In plants, Cu deficiency typically manifests as a potential disease that can ultimately affect gene expression changes that regulate various biological processes as well as root and leaf morphology [134,135,136]. The average Cu content in the plant dry weight is approximately 10 µg g^−1^/DW, and the critical Cu deficiency is generally between 1 µg g^−1^ and 5 µg g^−1^/DW [135,137,138]. In plants, Cu deficiency leads to growth retardation, increased senescence, and reduced productivity [98,136,139]. This may be caused by damage to the electron transport chain, leading to disruptions in photosynthesis and respiration [97]. This can also result in reduced plastoquinone synthesis, unsaturated C_18_ fatty acid content, isopropyl lipid synthesis, decreased CO_2_ fixation, and sensitivity to environmental stress [140,141]. Photosynthesis is the primary target of Cu deficiency [97]. Cu deficiency leads to a reduction in plastocyanin formation, which affects PSI electron transport, thereby reducing the net photosynthetic rate [53,141]. It has been suggested that severe Cu deficiency in plants can cause the disintegration of PSII complexes, directly altering the thylakoid structure and promoting the degradation of pigments (chlorophyll and carotenoid) [136,140,142]. Additionally, because CSD function is severely limited by Cu deficiency, it may lead to oxidative stress [143,144]. Owing to the poor migration rate of Cu in the phloem, the typical symptoms of Cu deficiency first appear in young leaves. These symptoms manifest as chlorosis at the top of the young leaves, leaf edge curling, leaf deformity, or even necrosis [98,139].

In *Arabidopsis*, Cu deficiency reduces the expression of genes involved in early anther development, leading to abnormal anther development and delayed flowering [99,145]. Furthermore, a variety of crop species, such as wheat, rice, oats, barley, sweet corn, and sunflower, have been used to demonstrate that Cu deficiency has a greater effect on reproduction than on vegetative growth, leading to reduced grain/seed yields [98]. Decreased fertility in Cu-deficient crops results from multiple factors, including stunted stigmas, reduced pollen fertility, and reduced pollination capacity [36]. However, compared with other micronutrients (including Fe, Mn, and Zn), there are few studies on the effects of Cu deficiency on plant responses to environmental stresses such as temperature, drought, and pathogen invasion (Figure 1).

### 4.2. Cu Toxicity

Owing to the redox properties of Cu ions, excess Cu accumulated in cells can have a toxic effect on plants. Toxic Cu concentrations vary significantly depending on plant species and genotypes, leading to significant differences in Cu tolerance within and among species [146,147]. Evidence suggests that photosynthesis is an early target of Cu toxicity [148]. After exposure to Cu stress, plants often exhibit a decrease in chlorophyll content and net photosynthetic rate [149,150]. The reduction in chlorophyll biosynthesis may be related to the structural damage of the photosynthetic apparatus on the thylakoid membrane under Cu stress and the interference of Cu on chlorophyll organization [151,152]. Some researchers have found that Cu has an inhibitory effect on both photosystems on the thylakoid membrane [8,153,154]. However, compared with PSI, PSII is more sensitive to Cu toxicity [8,153,154]. High concentrations of Cu can damage the non-heme type Fe^2+^ and cytochrome b_559_ on the PSII acceptor side, as well as the Mn cluster and external proteins of the oxygen evolution complex on the donor side. This damage can ultimately lead to impaired electron transfer and a reduced photosynthetic rate [155,156,157,158]. High concentrations of Cu can lead to structural damage to thylakoids, alter their lipid composition [159], induce lipid peroxidation [154], inhibit photophosphorylation [160], and reduce the efficiency of ribulose-1,5-bisphosphate carboxylase/oxygenase [161].

As a redox-active metal, excess Cu induces an increase in cellular ROS concentrations, such as hydrogen peroxide (H_2_O_2_), superoxide (O_2_^•−^), and hydroxyl radicals (^•^OH), and causes oxidative stress directly or indirectly by affecting cellular metabolic processes [162]. Evidence suggests that lipid peroxidation is an indicator of oxidative stress in plants [163]. In cells, the membrane system is the primary target of heavy metal poisoning. Excessive Cu also alters membrane lipids, affecting their total quantity, composition, and saturation levels [164]. In addition, ROS generated by Cu stress can attack hydrogen atoms in fatty acid chains (such as linoleic acid) in plasma and organelle membranes, leading to the generation of aldehydes and lipid free radicals [165,166]. These reactions can cause membrane lipid peroxidation, increased membrane permeability, massive extravasation of cellular contents, and cell death [167]. Some evidence suggests that, although H_2_O_2_ and O_2_^•−^can initiate lipid peroxidation, only ^•^OH is reactive enough to cause lipid peroxidation in the presence of Fe and Cu [168].

Another common effect of Cu toxicity on plants is the reduced absorption and homeostasis of other mineral nutrients [146]. Some evidence suggests that exposure to high concentrations of Cu reduces plant uptake of macronutrients, such as calcium (Ca), potassium (K), magnesium (Mg), and phosphorus (P), and trace elements, such as Fe, Mn, or Zn, or alters the distribution of these elements in roots and shoots [169,170,171,172]. High concentrations of Cu can disrupt the normal functions of certain ions during cell metabolism. Some researchers believe that Cu competes with the binding of -SH residues, thereby replacing cofactors (such as Mg^2+^, Zn^2+^, or Fe^2+^) in photosynthetic pigments or proteins in chloroplasts. Cu alters the ionic composition of photosynthetic pigments or proteins, making them unstable or inactive, leading to their degradation, and consequently affecting photosynthetic activity [49]. For example, Cu ions replace Mg^2+^ in chlorophyll molecules, resulting in a reduction in the chlorophyll content [173]. Excess Cu also reduces nitrate absorption, thereby inhibiting the growth of rice seedlings [174].

Symptoms of Cu toxicity vary widely among plant species and depend on the Cu content, duration, and developmental and physical stages of the plant [146,147]. Seed germination is the most sensitive physiological process to Cu stress in plants. Even a low dose of Cu can reduce the seed germination rate, inhibit bud growth, and impede root formation [175,176]. During plant growth, the root system plays an important role in absorbing nutrients from the soil and in responding to abiotic stress [177]. Therefore, the response of plants to Cu toxicity first occurs in the roots. High concentrations of Cu in the soil inhibit and damage root growth, leading to reduced absorption of nutrients and moisture by the roots. Under Cu stress, the typical symptoms of roots include root discoloration, reduced root hair proliferation, reduced primary root elongation, and severe deformation of the root structure [49]. Some evidence suggests that reduced root growth is often associated with the rupture of epidermal cells and root cell membranes and the thickening and rupture of the root cuticle [178,179]. However, the mechanism by which Cu stress affects root physiological processes to inhibit root development and the recognition of Cu stress signals remain unclear. It has been suggested that Cu ions may affect root growth and development by altering root meristem cell viability and mitotic activity, regulating plant hormones such as auxin and cytokinin, and influencing lignin deposition in the root system [180].

Another common and major symptom of plant Cu toxicity is leaf chlorosis, which often results in milky or white spots or lesions [181]. As Cu exposure increases, necrotic areas appear at the leaf tips and edges, whereas leaf area, diameter, and length decrease [182,183]. In acute Cu poisoning, leaves may wilt before eventually becoming necrotic [183]. Furthermore, Cu toxicity ultimately leads to reduced plant biomass and grain yields, which may pose serious health risks to humans through bioaccumulation in the food chain [7,149,184] (Figure 1).

## 5. Cu Transport in Plants

Cu is an essential micronutrient for plant growth and development, and its absorption by plants is an active transport process [185]. The cell wall is the first barrier for Cu ions to enter plant root cells and contains many macromolecular substances (including amino, phosphoric acid, hydroxyl, carboxyl, and hydroxyl) [186]. These coordination groups can undergo various physical or chemical reactions with positively charged Cu ions, adsorb excess Cu ions on the cell wall, and inhibit the entry of Cu ions into protoplasts. This process reduces the toxic effects of excessive Cu ions on plant cells [186,187].

Cu passes through the cell wall and forms various complexes with different amino acids and proteins within the cell. It is then transported to various tissues and organs through the xylem and phloem. At the tissue level, Cu transport mainly involves root absorption, vacuole sequestration, xylem and phloem loading, and node distribution and redistribution [188,189]. At the cellular level, the Cu transporters identified in plants can be divided into two categories: those responsible for transporting extracellular Cu to the intracellular space (absorption transporters) and those responsible for transporting intracellular Cu to the extracellular space or organelles (efflux transporters) [190]. In plant cells, Cu is maintained in a relatively balanced state through the coordinated regulation of these two types of Cu transporters. This balance ensures normal growth and development of plants and avoids the symptoms of Cu poisoning [188,191]. To date, several Cu transporters, including the high-affinity Cu absorption transporters CTR/COPT, P1B-ATPase/heavy metal ATPase, zinc–iron transporter ZIP, yellow stripe-like protein YSL, and Cu chaperones, have been identified and functionally characterized in monocotyledonous and dicotyledonous plants [192].

The COPT family of transporters consists of three transmembrane domains (TMDs) that contain His and/or Met domains, and the conserved motifs MXXXM and GXXXXG are associated with TMD2 and TMD3 [193]. According to the number of domains, the six COPT members reported in *Arabidopsis thaliana* can be divided into three categories. The first class consists of three Met- and His-rich domains: AtCOPT1, AtCOPT2, and AtCOPT6. These proteins exhibit strong affinity and transport activity for Cu, which is considered the primary reason for the ability of Cu to enter and exit cells [13]. The second type of members is AtCOPT3 and AtCOPT5, which have low binding capacities for Cu and cannot effectively transport Cu ions. Interestingly, the third class is AtCOPT4, which lacks the Met residues and the MXXXM motifs required for Cu ion transport. However, this protein can assist in Cu transport in plants by interacting with other COPT proteins [194].

P_1B_-ATPase, also known as heavy metal-transporting ATPase (HMA), consists of eight genes in *Arabidopsis thaliana* (AtHMA1-AtHMA8) and nine genes in rice (OsHMA1-AtHMA9) [195]. The HMA family includes members of all organisms. This class of proteins is not specific to metals, so the family can be divided into two subclasses: Cu subclass P_1B_-ATPase and Zn subclass P_1B_-ATPase [195]. The Cu subclasses in Arabidopsis include AtHMA5-AtHMA8, whereas they consist of OsHMA4-OsHMA9 in rice.

ZIP protein family members are primarily responsible for the transport of metal ions (including Zn, Cu, Fe, Mn, and Cd) into the cytoplasm and play an important role in maintaining the ionic balance of the cell [196]. At present, >100 ZIP proteins have been identified and are widely distributed in fungi, bacteria, animals, and plants; however, the functions of most genes have not been studied. Arabidopsis contains 11 ZIP (AtZIPl-AtZIP11) transporters, whereas rice contains 17 ZIP (OsZIP1-OsZIP17) transporters [25].

The YSL protein family primarily participates in the transport of metal elements in grasses, facilitating long-distance transport of nicotianamine (NA) and iron carriers within plants [197]. Eight members of the YSL family (AtYSL1-AtYSL8) have been identified in Arabidopsis, and 18 members of rice (OsYSL1-OsYSL18).

Cu chaperones are a class of small, low-molecular-weight metal receptor proteins that are widely present in various biological cells and contain a Cu ion-binding domain. This domain is responsible for binding Cu ions and delivering them to specific transporters for Cu transport to individual organelles [198]. Cu chaperones also play a role in preventing the toxic effects of free Cu ions on cells by inhibiting Cu binding to its intracellular components. For example, the expression level of antioxidant protein 1 (AtATX1) in Arabidopsis is upregulated by Cu-induced stress, and overexpression of *AtATX1* significantly enhances the accumulation and tolerance of Arabidopsis to Cu [199].

### 5.1. Cu Uptake from Soil and Translocation to Shoots

**Figure 2:**
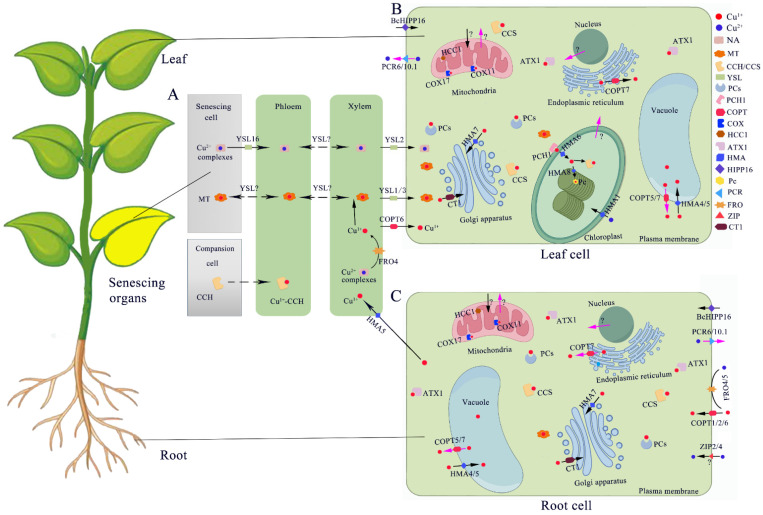
Overview of Cu transport mechanisms in plants. (**A**) Tissue specificity of Cu transporters, chaperones, and complexes. (**B**,**C**) subcellular localization of Cu transporters, chaperones, and complexes in root or leaf cells. In (**B**,**C**), black arrows indicate import into the cytosol, and purple arrows indicate export out of the cytosol. Proteins that undergo subcellular localization are only shown in yeast but not in plants and are indicated by a question mark. ZIP, Zn-regulated transporter; COPT, Copper transporter protein; FRO, Ferric reductase oxidase; HMA, Heavy metal ATPase; PCR, Plant cadmium resistance; PCH, Plas-tid chaperone; ATX1, Antioxidant protein; COX, Cytochrome coxidase; PCs, Phytochelatins; CCS/CCH, Cu chaperone; MTs, Metallothioneins; HCC1, the Arabidopsis homolog of the yeast mitochondrial copper chaperone SCO1; Pc, Plastocyanin; CT, MFS-type Cu trans-porter; YSL, Yellow stripe like; HIPP16, Heavy metal-associated isoprenylated plant protein 16.

The mechanism used to obtain Cu from the soil is similar to the reduced Fe absorption mechanism in dicotyledonous sand and non-grass monocotyledonous plants (Figure 2). For example, in Arabidopsis, roots are acidified by H^+^-ATPase, and Fe^3+^ is reduced to soluble Fe^2+^ using FRO2 localized to the plasma membrane [200]. Fe^2+^ is then absorbed by the high-affinity Fe transporter, IRT1, in the plasma membrane [201]. Similar to Fe, Cu is reduced on the root surface of Arabidopsis by two Fe reductase enzymes, FRO4 and FRO5, which are activated in the absence of Cu [202]. The reduced Cu^+^ is then transported into the cell by the transporters AtCOPT1/2 and ZIP2/4 [203,204]. AtCOPT1 is mainly expressed in root tips and localized to the plasma membrane, which mediates Cu uptake by roots from the soil. In the absence of Cu, the expression of *AtCOPT1* is upregulated, enhancing the ability of roots to obtain Cu from the soil [203]. During the absorption of Cu, OH is produced, which binds to non-selective cation channels on the plasma membrane and opens Ca ion channels. This process promotes the growth and development of roots [205]. Similar to AtCOPT1, the expression of *AtCOPT2* is also induced by Cu deficiency. Interestingly, when P is deficient in plants, AtCOPT2 transmits Cu to Cu-related proteins in response to low P signals by participating in P signal transduction [32]. In the case of Cu restriction, the expression of *ZIP2* and *ZIP4* is induced, whereas that of *ZIP4* is inhibited in the presence of excess Cu [206]. However, recent studies have been partially contradictory because Cu has been reported to overregulate *ZIP4* expression. In addition, because *ZIP4* expression is also correlated with Zn levels, there may be crosstalk in plant absorption of Cu and Zn [25].

In monocotyledonous plants, most members of the COPT family have not been well studied except COPT7. The expressions of *OsCOPT1*, *OsCOPT5*, and *OsCOPT7* were upregulated under Cu-deficient conditions. OsCOPT1 and OsCOPT5 synergistically mediate low-affinity Cu transport in yeast *mpy17* mutants by interacting with another protein, XA13, in rice [207]. These three proteins synergistically mediate Cu transport in rice. OsCOPT2, OsCOPT3, and OsCOPT4 physically interact with OsCOPT6, which can compensate for the absorption of Cu by the yeast mutants *scctr1* and *scctr3* [208]. However, OsCOPT7 could also compensate for Cu absorption in the yeast mutants. Recently, it has been proposed that OsCOPT7 is a Cu-exporting protein found in the vacuoles and endoplasmic reticulum [33]. It physically interacts with OsATX1 and plays an important role in regulating the root-to-shoot translocation of Cu in rice [33].

Once Cu is absorbed by the roots, it must be translocated to the shoot. In rice, *OsHMA5*, which is located in the plasma membrane, is responsible for the transport of Cu from the pericycle to the xylem through its interaction with OsATX1 [34]. Mutants of *oshma5* reduce the transport of Cu from roots to stems and seeds, consequently increasing the Cu concentration in roots [34]. Furthermore, OsATX1 interacts with OsHMA4, OsHMA6, and OsHMA9 to control the root-to-shoot translocation of Cu and its delivery to the developing tissue of rice [199]. Similarly, *AtHMA5* is mainly expressed in the roots, and its expression is upregulated by excessive Cu. The *athma5* mutants are more sensitive to Cu and accumulate higher levels of Cu in their roots. It has been suggested that *athma5* plays a detoxification role in the roots by translocating Cu to the xylem [18]. However, the tissue/organ-specific expression of *AtATX1* and the patterns of Cu accumulation in different tissues and organs remain unknown. Therefore, it is difficult to determine whether *AtATX1* affects Cu transport and distribution in plants through interactions with P_1B_-type ATPase.

During long-distance transport of Cu in the vascular systems of dicotyledonous plants, Cu^+^ may be oxidized to Cu^2+^ to facilitate transportation to the aboveground parts. Cu^2+^ binds to specific metal chelates in the xylem and phloem, such as metallothionein (MT), niacinamide, and 2′-deoxylyconic acid (DMA) [209]. To date, AtYSL2/AtYSL3 proteins have been well studied in vascular tissues. Both proteins are located on the lateral plasma membrane of the xylem parenchyma cells [23]. AtYSL2 transports Cu–NA and Fe–NA complexes, whereas overexpression of *AtYSL3* significantly increases the accumulation of Cu in leaves [210]. In Arabidopsis, these two transporters are involved in the lateral movement of Cu in root and stem vascular bundles. In rice, it has been found that OsYSL16 is not only involved in Fe transport from the roots to the aboveground parts but is also responsible for transporting Cu–NA complexes in vegetative organs to new tissues and seeds through the phloem. This process facilitates the distribution of nutrients from vegetative organs to reproductive organs [211]. It has been documented that the transport of Cu–NA from old leaves to new leaves and from flag leaves to spikelets is significantly reduced in *osysl16* knockout lines. Moreover, additional Cu levels can enhance the germination rate of mutants. In Arabidopsis, a protein known as AtCOPT6 has also been reported to participate in Cu remobilization and redistribution from green tissues to reproductive organs [16].

### 5.2. Intracellular Transport of Cu

It has been reported that the cytoplasm of eukaryotic cells is essentially free of Cu; Cu ions are bound to metal partners and transferred to various Cu-dependent enzymes and different organelles (such as the mitochondria and chloroplasts) or sequestered in vacuoles, exoplasmic bodies, or expelled out of the cell to eliminate excess Cu in the cytoplasm [8] (Figure 2).

#### 5.2.1. Vacuoles as Cu Stores

Vacuoles are the primary storage organelles in cells that store nutrients, including essential metals such as Cu, Zn, and Mn, and toxic elements, including nonessential metals such as cadmium and lead [212]. Therefore, vacuoles play an important role in the maintenance of intracellular metal ion homeostasis through the sequestration of metal ions during cell detoxification and the influx of metal ions in the vacuoles under nutrient-deficient conditions. AtCOPT5 is a Cu transporter located in the vacuole membrane that transfers Cu stored in the vacuole to the cytoplasm to maintain normal plant growth under Cu-deficient conditions [15]. In rice, OsHMA4 is involved in sequestering Cu in the vacuoles of root cells. Mutations in *OsHMA4* result in increased Cu transport from the roots to the shoots, leading to increased Cu accumulation in the grains [212,213]. Cu transporters in tonoplasts have also been reported in other species. For example, both CsHMA5.1 and CsHMA5.2 mediate the transport of Cu from the cytoplasm to the vacuole. The transcript of CsHMA5.1 is root-specific and unaffected by Cu concentration, whereas the transcript of CsHMA5.2 is significantly upregulated in the presence of excess Cu [214]. Under the toxic effects of Cu, CsHMA5.2 can increase the accumulation of Cu in the vacuoles of cucumber cells [214].

#### 5.2.2. Cu Accumulation in Chloroplasts

Proteins in chloroplasts and mitochondria are involved in electron transport and redox processes, and Cu is required as a cofactor to perform these functions [215]. In the chloroplast, Cu is transported to plastocyanin, a mobile electron carrier that plays an important role in the synthesis of photoreactive ATP and NADPH [57]. In Arabidopsis, AtPCH1 and AtCCS are the main components responsible for Cu allocation to chloroplasts [216]. AtPCH1 transfers Cu from the cytoplasm to the intermembrane space, and then the metal to AtPAA1/AtHMA6. It mediates the transport of Cu to the chloroplast matrix, where metal ions bind to AtCCS and are transferred to AtCSD2 and AtPAA2/AtHMA8, which are located in the thylakoid membrane [19,21]. Finally, AtPAA2/AtHMA8 delivers Cu to plastocyanin in the thylakoid lumen. AtPAA2/AtHMA8 may also play a role in protecting thylakoids from Cu toxicity at high Cu concentrations [21].

#### 5.2.3. Cu Accumulation in Mitochondria

Although Cu is also important for mitochondrial function, the transport mechanism of Cu in plant cell mitochondria remains unclear compared with that in chloroplasts. However, the transport mechanism of Cu in the mitochondria of yeast has been well studied. It has been shown that the Cu-chaperone ScCOX17 (cytochrome c oxidase) delivers Cu to the Cu-chaperone proteins ScCOX11 and ScSCO1 in the mitochondrial membrane, which then loads Cu to the CuA and CuB centers [217]. They provide Cu for COX (Cu-dependent cytochrome c oxidase) subunits I and II, respectively. There are two homologues of ScCOX17 in Arabidopsis: AtCOX17-1 and AtCOX17-2. AtCOX17-1 is localized to the mitochondria and is involved in the transfer of Cu within the mitochondria [30]. In addition, two homologs of SCO1 in yeast have been found in Arabidopsis: AtHCC1 and AtHCC2. Although the two ATHCCS share a high degree of homology, the CXXXC motif and histidine residue critical for Cu binding are not conserved in HCC2, suggesting that the two proteins have different roles. AtHCC1 is upregulated under excess Cu and contains CXXXC motifs and specific histidine residues required for Cu binding [218]. In contrast, AtHCC2 lacks a Cu-binding motif and is therefore thought to be involved in redox regulation and the UV-B stress response rather than Cu transport within the mitochondria in plants [219]. AtCOX11, a homolog of the yeast ScCOX11, has also been identified in Arabidopsis and is localized to the mitochondria. Interference and overexpression of *AtCOX11* can affect COX activity and reduce pollen germination, indicating that COX11 plays a key role in Cu homeostasis in the mitochondria [220].

#### 5.2.4. Cu Homeostasis in Endomembranes

In Arabidopsis, cytoplasmic Cu binds to the Cu-chaperone protein CCH, which interacts with AtHMA7/AtRAN1 located in the Golgi apparatus to transport Cu to the intracavity of the vesicles, where Cu is integrated into the ethylene receptor protein ETR1 [221]. In Arabidopsis, the ETR1 receptor contains five subunits that mediate its response to ethylene. They consist of two domains: an N-terminal transmembrane domain, which is involved in Cu and ethylene binding, and a cytoplasmic C-terminal domain, which is involved in signal transduction [222]. After binding to the transmembrane domain, Cu is required as a cofactor to deliver it to the receptor and thus bind ethylene with a high affinity [222]. The transfer of Cu to ethylene receptors may be catalyzed by the ATPase AtHMA7/AtRAN1. Loss of function of AtRAN1/AtHMA7 in Arabidopsis leads to yellowing of seedlings and a constitutive ethylene reaction, resulting in severe growth inhibition or death [223]. In addition to delivering Cu to proteins that require it, the secretory pathway-targeted HMA7/RAN1 transporter may also play a role in maintaining whole-cell Cu homeostasis, thereby enhancing plant tolerance to excess Cu [221]. In addition to the P_1B_-type Cu transport ATPase, members of the major facility superfamily (MFS) family are also involved in Golgi-mediated Cu homeostasis in plant cells. TaCT1 is a transporter from the common wheat MFS family that is located in the Golgi apparatus and participates in Cu transport. In contrast to white grass and AtRAN1/AtHMA7, the transcriptional levels of wheat TaCT1 were downregulated during excess Cu and upregulated during Cu deficiency. This suggests that Cu transporters in the MFS family in plants help maintain cellular Cu homeostasis by facilitating Cu uptake under Cu-deficient conditions [42]. In addition to the Golgi apparatus, the endoplasmic reticulum also plays an important role in intracellular Cu homeostasis. Recently, it was proposed that OsCOPT7 is involved in transporting Cu from the endoplasmic reticulum to the cytoplasm. It physically interacts with OsATX1 to regulate the root-to-shoot translocation of Cu in rice [33].

#### 5.2.5. Cu Homeostasis by Efflux Pathway

In the case of Cu poisoning, plant cells maintain Cu homeostasis in the cytoplasm, primarily by expelling Cu^2+^ from the plasma membrane. AtHMA5 interacts with the Cu partner ATX1-like to transport excess Cu to the plasma membrane, where it is excreted. AtHMA5 can transport Cu to exosomal oxidase and laccase, both of which are involved in cell wall formation [18]. In rice, OsHMA5 and OsHMA9 participate in Cu expulsion and reduce the accumulation of Cu in cells [35]. In addition, BnPCR10.1 from *Brassica napus* and SlPCR6 from *Salix linearistipularis* enhance Cu efflux from cells [44,224].

## 6. Cu Homeostasis Is Mediated by the Regulation of Transcription Factors

**Figure 3:**
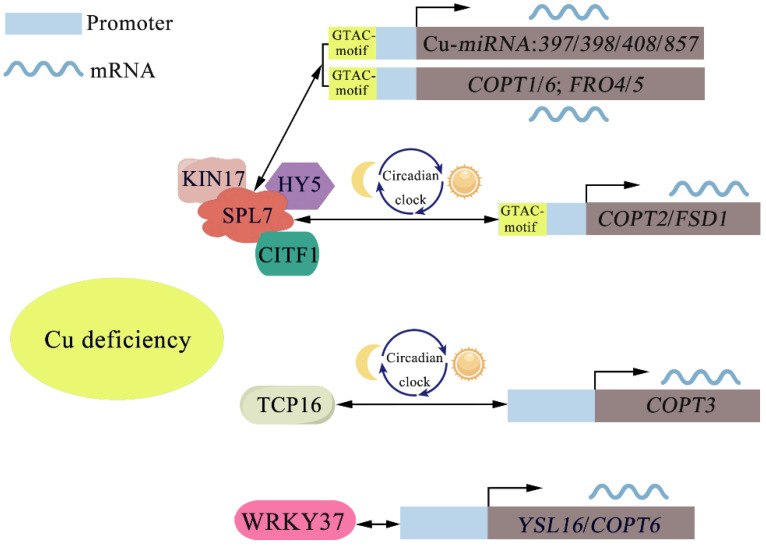
Transcriptional regulatory mechanisms in plants responding to copper deficiency. Black straight arrows indicate transcription activation; black curved arrows indicate the initiation of transcription. SPL7, SQUAMOSA promoter binding protein-like 7; KIN17, Kin17 DNA and RNA binding protein; HY5, elongated hypocotyl 5; CITF1, Cu-deficiency-induced transcription factor 1; TCP, Teosinte branched1–cycloidea–proliferating cell factor1.

In recent years, the significance of transcription factors in maintaining Cu homeostasis has been gradually revealed. SPL7 (SQUAMOSA promoter binding protein-like 7) is a well-known core transcription factor involved in sensing Cu deficiency signals [225,226]. Under Cu deficiency conditions, SPL7 can bind to the GTAC motif within the Cu response element in the promoter region of Cu-responsive genes. This binding activates the Cu-metal reductase FRO4/5 and the Cu transporter COPT1/2/6 expression of a high-affinity Cu uptake system [202,206,226,227]. Some evidence suggests that SPL7 needs to directly interact with multiple proteins, such as KIN17, HY5 (elongated hypocotyl 5), and CITF1 (Cu-deficiency-induced transcription factor), to promote its binding to the Cu response element of the target gene [99,227,228]. Furthermore, under conditions of Cu deficiency, SPL7 inhibits the expression of other Cu-responsive genes by activating miRNAs to deliver Cu to the essential Cu-containing proteins. These miRNAs are called Cu-miRNAs and include *miR397*, *miR398*, *miR408*, and *miR857* [105,229]. They target the mRNA of less important Cu-containing proteins, such as Cu/Zn superoxide dismutase and plantain cyanin. Evidence suggests that Cu homeostasis in plant cells is regulated by circadian rhythms. It has been proposed that SPL7 regulates the expression of *COPT2* and Fe superoxide dismutase 1 (FSD1) through circadian rhythms in response to cellular demands for Cu [230] (Figure 3). In addition, the circadian clock protein TCP16 (Teosinte branched 1, cycloidea proliferation cell factor 1) can bind to the *COPT3* promoter and down-regulate its expression, thereby reducing the Cu content and affecting pollen development [231] (Figure 3). Other well-known transcription factor families, such as WRKY, are also directly involved in regulating cellular Cu homeostasis. Recent studies have shown that it can induce transcription of *OsWRKY37*, which binds to promoters of *OsCOPT6* and *OsYSL16*, thereby positively regulating their expression [232] (Figure 3). However, some transcription factors affect Cu absorption and distribution indirectly. FIT (Fer-like iron deficiency-induced transcription factor) and four bHLH Ib transcription factors (bHLH38, bHLH39, bHLH100, and bHLH101) directly bind to the promoters of *COPT2*, *FRO4*, and *FRO5* to enhance Cu absorption and alleviate Fe-deficiency symptoms, contributing to the mutual balance between Cu and Fe [233]. PIF3/4/5 (phytochrome-interacting factors) directly binds to the promoters of *miR408* to regulate dark-induced senescence in Arabidopsis leaves by facilitating intracellular Cu redistribution [234]. The findings highlight the enhancement of Cu uptake by plants and their subsequent transportation to the soil and reproductive organs, thereby promoting crop growth and increasing crop yield.

## 7. Conclusions and Future Perspective

The relevance of Cu as a plant micronutrient remains largely underestimated. It is often believed that plant cells have low physiological requirements for Cu and that they absorb Cu in amounts that meet their metabolic requirements. However, in natural and agricultural environments, Cu availability is limited by factors such as soil type and its physical and chemical properties. Undetected and unrepaired latent Cu deficiencies can impair plant growth and hinder sustainable and productive agriculture. Cu deficiency can be remedied by applying Cu-based fertilizers, which may lead to Cu accumulation in the soil. However, with industrial production (such as mineral development and chemical manufacturing) and agricultural activities (such as the use of Cu-based agricultural chemicals), soil Cu pollution has become increasingly serious. Excessive Cu accumulation in cells inhibits plant root growth and leaf photosynthesis, thereby severely limiting plant productivity. Several measures have been used to mitigate the toxicity of Cu in soil to plants, including the application of soil amendments such as lime or fly ash, adding organic fertilizers, and limiting the use of Cu-based pesticides. However, the choice and availability of these remedies depend on various factors, including availability, cost, and duration of effectiveness.

Increasing evidence suggests that using the endogenous metal transport mechanism in plants could be a key measure to address issues related to metal deficiency or toxicity. For example, through gene editing, the functions of high-affinity Cu uptake and transport proteins in plant roots are suppressed to reduce Cu uptake by plants growing in Cu-polluted areas. Others improve plant tolerance to metals by sequestering metal ions into cellular compartments such as vacuoles. However, a prerequisite for implementing these measures is to fully understand the regulatory mechanisms of Cu transport between tissues and cellular homeostasis. Key questions include, for example, what is the complete molecular pathway through which Cu, taken up by roots from the soil, is transported to the vascular bundles and loaded or unloaded in the xylem or phloem? In grasses, such as rice, certain nodes play a role in the transport of metal ions from roots to aboveground parts and the redistribution of aboveground parts. Given that the aboveground parts of plants have a limited demand for Cu, how do these nodes precisely regulate Cu transport? What transport proteins are involved in these processes? Furthermore, at the cellular level, is the trans-Golgi network-mediated vesicle secretion mechanism involved in intracellular Cu detoxification, in addition to vacuolar sequestration?

Other challenges to be solved include plant sensing and signaling of Cu status. This review highlighted some transcription factors, such as SPL7 and OsWRKY37, that regulate the expression of related genes in response to Cu deficiency in plants. However, the signaling mechanisms underlying the response of plants to Cu stress remain unclear.

Future research should prioritize addressing these scientific questions and investigating the regulatory mechanisms of plants in response to Cu deficiency or stress. Because Cu deficiency and toxicity are both limiting factors for agricultural crop productivity, a better understanding of Cu homeostasis in plants, combined with related biological breeding, will enhance the Cu utilization efficiency and/or Cu tolerance of crops in alkaline or Cu-contaminated soils in the future.

## Figures and Tables

**Table 1:** Copper uptake and transport genes in different species.

Species	Gene	Subcellular Localization	Tissue Expression	Reference
Arabidopsis (*Arabidopsis thaliana*)	*AtCOPT1*	Plasma membrane	Most tissues, roots, and reproductive tissues	[13]
	*AtCOPT2*	Plasma membrane	Most tissues and roots	[13]
	*AtCOPT3*	Plasma membrane	Reproductive tissues	[13]
	*AtCOPT5*	Prevacuolar compartment and tonoplast	Most tissues, roots, and reproductive tissues	[14,15]
	*AtCOPT6*	Plasma membrane	Reproductive tissues, xylem, and phloem vascular tissues	[16]
	*AtHMA1*	Chloroplast envelope	Green tissues	[17]
	*AtHMA5*	Plasma membrane	Roots and flowers	[18]
	*AtHMA6*	Chloroplast	shoots	[19]
	*AtHMA7*	*Trans-Golgi* network?	Roots and flowers	[20]
	*AtHMA8*	Thylakoid membrane	Aboveground	[21]
	*AtYSL1*	Plasma membrane	Most tissues and roots	[22]
	*AtYSL2*	Plasma membrane	Most tissues, roots, and stems	[23]
	*AtYSL3*	Plasma membrane	Young leaves, roots, and stems	[22]
	*AtZIP2*	Plasma membrane	Roots	[24,25]
	*AtZIP4*	Plasma membrane	Roots	[24,26]
	*AtCCH*	Cytosol	Stems and vascular tissues	[27]
	*AtATX1*	Cytosol	——	[28]
	*AtCCS*	Cytosol and chloroplast	Stems, flowers and leaves	[29]
	*AtCOX17–1*	——	——	[30]
	*AtCOX17–2*	——	——	[30]
Rice (*Oryza sativa*)	*OsCOPT1*	Plasma membrane	Most tissues, roots, and stems	[31]
	*OsCOPT2*	Plasma membrane	Most tissues and roots	[32]
	*OsCOPT7*	Tonoplast and endoplasmic reticulum	Most tissues and roots	[33]
	*OsHMA5*	Plasma membrane	Xylem of vascular bundles at the nodes, pedicels, and petioles	[34]
	*OsHMA9*	Plasma membrane	Xylem and phloem vascular tissues	[35]
	*OsYSL16*	Plasma membrane	Phloem and vascular tissues of the roots, stems, and leaves	[36]
Medicago (*Medicago truncatula*)	*MtZIP4*	——	Roots and leaves	[37]
	*MtCOPT1*	Plasma membrane	Roots	[38]
	*MtCOPT3*	——	Nodules	[38]
	*MtCOPT4*	——	Roots	[38]
	*MtCOPT5*	——	Roots	[38]
	*MtCOPT8*	——	Roots, xylem, and phloem vascular tissues	[38]
Grape (*Vitis vinifera*)	*VvCTr1*	Tonoplast	Xylem and phloem vascular tissues, leaves, and roots	[39]
	*VvCTr2*	——	——	[40]
	*VvCTr8*	——	——	[40]
Soybean (*Glycine max*)	*GmHMA8*	Thylakoid membrane	Leaves	[41]
Wheat (*Triticum aestivum*)	*TaCT1*	Golgi apparatus	Xylem and phloem vascular tissues, roots, and grains	[42]
Rape (*Brassica napus*)	*BnHMA1*	——	Leaves	[43]
	*BnCOPT2*	——	Roots	[43]
	*BnPCR10.1*	Plasma membrane	Most tissues	[44]
Barley (*Hordeum vulgare*)	*HvHMA1*	Chloroplast envelopes	Leaves and seeds	[45]
	*HvYSL2*	——	Stems, and young leaves	[46]
Peanut (*Arachis hypogaea*)	*AhYSL3.1*	Plasma membranes	Roots, stems, young leaves, and old leaves	[47]
	*AhYSL3.2*	——	Roots, stems, and leaves	[47]

## Data Availability

Not applicable.

## References

[1] Altarelli M., Ben-Hamouda N., Schneider A., Berger M.M. (2019). Copper deficiency: Causes, manifestations, and treatment. Nutr. Clin. Pract..

[2] Gaetke L.M., Chow-Johnson H.S., Chow C.K. (2014). Copper: Toxicological relevance and mechanisms. Arch. Toxicol..

[3] Schulten A., Krämer U. (2018). Interactions between copper homeostasis and metabolism in plants. Prog. Bot..

[4] White P.J., Broadley M.R. (2009). Biofortification of crops with seven mineral elements often lacking in human diets–iron, zinc, copper, calcium, magnesium, selenium and iodine. New Phytol..

[5] Shotyk W. (2020). Natural and anthropogenic sources of copper to organic soils: A global, geochemical perspective. Can. J. Soil Sci..

[6] Wei X., Hao M., Shao M. (2007). Copper fertilizer effects on copper distribution and vertical transport in soils. Geoderma.

[7] Fagnano M., Agrelli D., Pascale A., Adamo P., Fiorentino N., Rocco C., Pepe O., Ventorino V. (2020). Copper accumulation in agricultural soils: Risks for the food chain and soil microbial populations. Sci. Total Environ..

[8] Mir A.R., Pichtel J., Hayat S. (2021). Copper: Uptake, toxicity and tolerance in plants and management of Cu-contaminated soil. Biometals.

[9] Aragón M.S., Nakamaru Y.M., García-Carmona M., Garzón F.J.M., Peinado F.J.M. (2019). The role of organic amendment in soils affected by residual pollution of potentially harmful elements. Chemosphere.

[10] Joris H.A., da Fonseca A.F., Asami V.Y., Briedis C., Borszowskei P.R., Garbuio F.J. (2012). Heavy metals adsorption after surface lime in a Rhodic Hapludox under no-tillage system. Rev. Ciênc. Agrôn..

[11] Lee D.-S., Lim S.-S., Park H.-J., Yang H.I., Park S.-I., Kwak J.-H., Choi W.-J. (2019). Fly ash and zeolite decrease metal uptake but do not improve rice growth in paddy soils contaminated with Cu and Zn. Environ. Int..

[12] Burkhead J.L., Gogolin Reynolds K.A., Abdel-Ghany S.E., Cohu C.M., Pilon M. (2009). Copper homeostasis. New Phytol..

[13] Sanz A., Pike S., Khan M.A., Carrió-Seguí À., Mendoza-Cózatl D.G., Peñarrubia L., Gassmann W. (2019). Copper uptake mechanism of *Arabidopsis thaliana* high-affinity COPT transporters. Protoplasma.

[14] Garcia-Molina A., Andrés-Colás N., Perea-García A., del Valle-Tascón S., Peñarrubia L., Puig S. (2011). The intracellular *Arabidopsis* COPT5 transport protein is required for photosynthetic electron transport under severe copper deficiency. Plant J..

[15] Klaumann S., Nickolaus S.D., Fürst S.H., Starck S., Schneider S., Ekkehard Neuhaus H., Trentmann O. (2011). The tonoplast copper transporter COPT5 acts as an exporter and is required for interorgan allocation of copper in *Arabidopsis thaliana*. New Phytol..

[16] Jung H.I., Gayomba S.R., Rutzke M.A., Craft E., Kochian L.V., Vatamaniuk O.K. (2012). COPT6 is a plasma membrane transporter that functions in copper homeostasis in *Arabidopsis* and is a novel target of SQUAMOSA promoter-binding protein-like 7. J. Biol. Chem..

[17] Boutigny S., Sautron E., Finazzi G., Rivasseau C., Frelet-Barrand A., Pilon M., Rolland N., Seigneurin-Berny D. (2014). HMA1 and PAA1, two chloroplast-envelope P_IB_-ATPases, play distinct roles in chloroplast copper homeostasis. J. Exp. Bot..

[18] Andrés-Colás N., Sancenón V., Rodríguez-Navarro S., Mayo S., Thiele D.J., Ecker J.R., Puig S., Peñarrubia L. (2006). The *Arabidopsis* heavy metal P-type ATPase HMA5 interacts with metallochaperones and functions in copper detoxification of roots. Plant J..

[19] Catty P., Boutigny S., Miras R., Joyard J., Rolland N., Seigneurin-Berny D. (2011). Biochemical characterization of AtHMA6/PAA1, a chloroplast envelope Cu (I)-ATPase. J. Biol. Chem..

[20] Baloun J., Nevrtalova E., Kovacova V., Hudzieczek V., Cegan R., Vyskot B., Hobza R. (2014). Characterization of the *HMA7* gene and transcriptomic analysis of candidate genes for copper tolerance in two *Silene vulgaris* ecotypes. J. Plant Physiol..

[21] Tapken W., Kim J., Nishimura K., van Wijk K.J., Pilon M. (2015). The Clp protease system is required for copper ion-dependent turnover of the PAA2/HMA8 copper transporter in chloroplasts. New Phytol..

[22] Waters B.M., Chu H.-H., DiDonato R.J., Roberts L.A., Eisley R.B., Lahner B., Salt D.E., Walker E.L. (2006). Mutations in *Arabidopsis yellow stripe-like1* and *yellow stripe-like3* reveal their roles in metal ion homeostasis and loading of metal ions in seeds. Plant Physiol..

[23] DiDonato R.J., Roberts L.A., Sanderson T., Eisley R.B., Walker E.L. (2004). *Arabidopsis* *Yellow Stripe-Like2* (*YSL2*): A metal-regulated gene encoding a plasma membrane transporter of nicotianamine–metal complexes. Plant J..

[24] Wintz H., Fox T., Wu Y.-Y., Feng V., Chen W., Chang H.-S., Zhu T., Vulpe C. (2003). Expression profiles of *Arabidopsis thaliana* in mineral deficiencies reveal novel transporters involved in metal homeostasis. J. Biol. Chem..

[25] Milner M.J., Seamon J., Craft E., Kochian L.V. (2013). Transport properties of members of the ZIP family in plants and their role in Zn and Mn homeostasis. J. Exp. Bot..

[26] Lee S., Lee J., Ricachenevsky F.K., Punshon T., Tappero R., Salt D.E., Guerinot M.L. (2021). Redundant roles of four ZIP family members in zinc homeostasis and seed development in *Arabidopsis thaliana*. Plant J..

[27] Mira H., Martínez-García F., Peñarrubia L. (2001). Evidence for the plant-specific intercellular transport of the *Arabidopsis* copper chaperone CCH. Plant J..

[28] Shin L.J., Yeh K.C. (2012). Overexpression of *Arabidopsis* ATX1 retards plant growth under severe copper deficiency. Plant Signal Behav..

[29] Peñarrubia L., Romero P., Carrió-Seguí A., Andrés-Bordería A., Moreno J., Sanz A. (2015). Temporal aspects of copper homeostasis and its crosstalk with hormones. Front. Plant Sci..

[30] Balandin T., Castresana C. (2002). AtCOX17, an *Arabidopsis* homolog of the yeast copper chaperone COX17. Plant Physiol..

[31] Navarro B.B., Del Frari B.K., da Cruz Dias P.V., Lemainski L.E., Mario R.B., Ponte L.R., Goergen A., Tarouco C.P., Neves V.M., Dressler V.L.J.P.P. (2021). The copper economy response is partially conserved in rice (*Oryza sativa* L.). Plant Physiol. Biochem..

[32] Perea-García A., Garcia-Molina A., Andrés-Colás N., Vera-Sirera F., Pérez-Amador M.A., Puig S., Peñarrubia L. (2013). *Arabidopsis* copper transport protein COPT2 participates in the cross talk between iron deficiency responses and low-phosphate signaling. Plant Physiol..

[33] Tang Z., Li Y.F., Zhang Z.H., Huang X.Y., Zhao F.J. (2024). OsCOPT7 is a copper exporter at the tonoplast and endoplasmic reticulum and controls Cu translocation to the shoots and grain of rice. Plant Cell Environ..

[34] Deng F., Yamaji N., Xia J., Ma J.F. (2013). A member of the heavy metal P-type ATPase OsHMA5 is involved in xylem loading of copper in rice. Plant Physiol..

[35] Lee S., Kim Y.-Y., Lee Y., An G. (2007). Rice P_1B_-type heavy-metal ATPase, OsHMA9, is a metal efflux protein. Plant Physiol..

[36] Zhang C., Lu W., Yang Y., Shen Z., Ma J.F., Zheng L. (2018). OsYSL16 is required for preferential Cu distribution to floral organs in rice. Plant Cell Physiol..

[37] AF L.-M. (2004). Identification and characterization of several new members of the ZIP family of metal ion in *Medicago truncatula*. Plant Mol. Biol..

[38] Senovilla M., Castro-Rodríguez R., Abreu I., Escudero V., Kryvoruchko I., Udvardi M.K., Imperial J., González-Guerrero M. (2018). *Medicago truncatula* copper transporter 1 (MtCOPT1) delivers copper for symbiotic nitrogen fixation. New Phytol..

[39] Martins V., Bassil E., Hanana M., Blumwald E., Gerós H. (2014). Copper homeostasis in grapevine: Functional characterization of the Vitis vinifera copper transporter 1. Planta.

[40] Martins V., Hanana M., Blumwald E., Gerós H. (2012). Copper transport and compartmentation in grape cells. Plant Cell Physiol..

[41] Bernal M., Testillano P.S., Alfonso M., del Carmen Risueno M., Picorel R., Yruela I. (2007). Identification and subcellular localization of the soybean copper P_1B_-ATPase GmHMA8 transporter. J. Struct. Biol..

[42] Li H., Fan R., Li L., Wei B., Li G., Gu L., Wang X., Zhang X. (2014). Identification and characterization of a novel copper transporter gene family *TaCT1* in common wheat. Plant Cell Environ..

[43] Billard V., Ourry A., Maillard A., Garnica M., Coquet L., Jouenne T., Cruz F., Garcia-Mina J.-M., Yvin J.-C., Etienne P. (2014). Copper-deficiency in *Brassica napus* induces copper remobilization, molybdenum accumulation and modification of the expression of chloroplastic proteins. PLoS ONE.

[44] Liu Y., Kong L., Gong C., Yang G., Xu E., Chen W., Zhang W., Chen X. (2023). Identification of plant cadmium resistance gene family in *Brassica napus* and functional analysis of BnPCR10. 1 involved in cadmium and copper tolerance. Plant Physiol. Biochem..

[45] Mikkelsen M.D., Pedas P., Schiller M., Vincze E., Mills R.F., Borg S., Møller A., Schjoerring J.K., Williams L.E., Baekgaard L. (2012). Barley HvHMA1 is a heavy metal pump involved in mobilizing organellar Zn and Cu and plays a role in metal loading into grains. PLoS ONE.

[46] Araki R., Murata J., Murata Y. (2011). A novel barley yellow stripe 1-like transporter (HvYSL2) localized to the root endodermis transports metal-phytosiderophore complexes. Plant Cell Physiol..

[47] Dai J., Wang N., Xiong H., Qiu W., Nakanishi H., Kobayashi T., Nishizawa N.K., Zuo Y. (2018). The *yellow stripe-like* (*YSL*) gene functions in internal copper transport in peanut. Genes.

[48] Migocka M., Malas K. (2018). Plant responses to copper: Molecular and regulatory mechanisms of copper uptake, distribution and accumulation in plants. Plant Micronutrient Use Efficiency.

[49] Shabbir Z., Sardar A., Shabbir A., Abbas G., Shamshad S., Khalid S., Murtaza G., Dumat C., Shahid M. (2020). Copper uptake, essentiality, toxicity, detoxification and risk assessment in soil-plant environment. Chemosphere.

[50] Cohu C.M., Pilon M. (2010). Cell biology of copper. Cell Biology of Metals Nutrients.

[51] Murphy J.M., Powell B.A., Brumaghim J.L. (2020). Stability constants of bio-relevant, redox-active metals with amino acids: The challenges of weakly binding ligands. Coord. Chem. Rev..

[52] Waldron K.J., Rutherford J.C., Ford D., Robinson N. (2009). Metalloproteins and metal sensing. Nature.

[53] Shikanai T., Müller-Moulé P., Munekage Y., Niyogi K.K., Pilon M. (2003). PAA1, a P-type ATPase of *Arabidopsis*, functions in copper transport in chloroplasts. Plant Cell.

[54] Schmidt S.B., Eisenhut M., Schneider A. (2020). Chloroplast transition metal regulation for efficient photosynthesis. Trends Plant Sci..

[55] Fedorov V.A., Kovalenko I.B., Khruschev S.S., Ustinin D.M., Antal T.K., Riznichenko G.Y., Rubin A.B. (2019). Comparative analysis of plastocyanin–cytochrome f complex formation in higher plants, green algae and cyanobacteria. Physiol. Plant.

[56] Yang X., Che Y., García V.J., Shen J., Zheng Y., Su Z., Zhu L., Luan S., Hou X. (2023). Cyclophilin 37 maintains electron transport via the cytochrome b6/f complex under high light in *Arabidopsis*. Plant Physiol..

[57] Höhner R., Pribil M., Herbstová M., Lopez L.S., Kunz H.-H., Li M., Wood M., Svoboda V., Puthiyaveetil S., Leister D. (2020). Plastocyanin is the long-range electron carrier between photosystem II and photosystem I in plants. Proc. Natl. Acad. Sci. USA.

[58] Kropat J., Gallaher S.D., Urzica E.I., Nakamoto S.S., Strenkert D., Tottey S., Mason A.Z., Merchant S.S. (2015). Copper economy in Chlamydomonas: Prioritized allocation and reallocation of copper to respiration vs. photosynthesis. Proc. Natl. Acad. Sci. USA.

[59] Howe C.J., Nimmo R.H., Barbrook A.C., Bendall D.S. (2016). Cytochrome c_6A_ of chloroplasts. Cytochrome Complexes: Evolution, Structures, Energy Transduction, Signaling.

[60] Chida H., Yokoyama T., Kawai F., Nakazawa A., Akazaki H., Takayama Y., Hirano T., Suruga K., Satoh T., Yamada S. (2006). Crystal structure of oxidized cytochrome c_6A_ from *Arabidopsis thaliana*. FEBS Lett..

[61] Weigel M., Varotto C., Pesaresi P., Finazzi G., Rappaport F., Salamini F., Leister D. (2003). Plastocyanin is indispensable for photosynthetic electron flow in *Arabidopsis thaliana*. J. Biol. Chem..

[62] Shosheva A., Donchev A., Dimitrov M., Kostov G., Toromanov G., Getov V., Alexov E. (2005). Comparative study of the stability of poplar plastocyanin isoforms. Biochim. Biophys. Acta, Proteins Proteomics.

[63] Dimitrov M.I., Donchev A.A., Egorov T.A. (1993). Twin plastocyanin dimorphism in tobacco. Biochim. Biophys. Acta Protein Struct. Mol. Enzymol..

[64] Yu J., Wang J., Lin W., Li S., Li H., Zhou J., Ni P., Dong W., Hu S., Zeng C. (2005). The genomes of *Oryza sativa*: A history of duplications. PLoS Biol..

[65] Rensing S.A., Ick J., Fawcett J.A., Lang D., Zimmer A., Van de Peer Y., Reski R. (2007). An ancient genome duplication contributed to the abundance of metabolic genes in the moss *Physcomitrella patens*. BMC Evol. Biol..

[66] Abdel-Ghany S.E. (2009). Contribution of plastocyanin isoforms to photosynthesis and copper homeostasis in *Arabidopsis thaliana* grown at different copper regimes. Planta.

[67] Pesaresi P., Scharfenberg M., Weigel M., Granlund I., Schröder W.P., Finazzi G., Rappaport F., Masiero S., Furini A., Jahns P. (2009). Mutants, overexpressors, and interactors of *Arabidopsis* plastocyanin isoforms: Revised roles of plastocyanin in photosynthetic electron flow and thylakoid redox state. Mol. Plant.

[68] Zhang Y., Yang Z., Zhang Z., Li Y., Guo J., Liu L., Wang C., Fan H., Wang B., Han G. (2023). Root hair development and adaptation to abiotic stress. J. Agric. Food Chem..

[69] Zhang J., Sun X. (2021). Recent advances in polyphenol oxidase-mediated plant stress responses. Phytochemistry.

[70] Boeckx T., Winters A.L., Webb K.J., Kingston-Smith A.H. (2015). Polyphenol oxidase in leaves: Is there any significance to the chloroplastic localization?. J. Exp. Bot..

[71] Garcia L., Welchen E., Gonzalez D.H. (2014). Mitochondria and copper homeostasis in plants. Mitochondrion.

[72] Llases M.E., Lisa M.N., Morgada M.N., Giannini E., Alzari P.M., Vila A.J. (2020). *Arabidopsis thaliana* HCC1 is a Sco-like metallochaperone for CuA assembly in Cytochrome c Oxidase. FEBS J..

[73] Wikström M., Gennis R.B., Rich P.R. (2023). Structures of the intermediates in the catalytic cycle of mitochondrial cytochrome c oxidase. Biochim. Biophys. Acta Bioenerg..

[74] Waller R.F., Keeling P.J. (2006). Alveolate and chlorophycean mitochondrial *cox2* genes split twice independently. Gene.

[75] Daley D.O., Adams K.L., Clifton R., Qualmann S., Millar A.H., Palmer J.D., Pratje E., Whelan J. (2002). Gene transfer from mitochondrion to nucleus: Novel mechanisms for gene activation from *Cox2*. Plant J..

[76] Soto I.C., Fontanesi F., Liu J., Barrientos A. (2012). Biogenesis and assembly of eukaryotic cytochrome c oxidase catalytic core. Biochim. Biophys. Acta Bioenerg..

[77] Mazurenko I., Adachi T., Ezraty B., Ilbert M., Sowa K., Lojou E. (2022). Electrochemistry of copper efflux oxidase-like multicopper oxidases involved in copper homeostasis. Curr. Opin. Electrochem..

[78] McCaig B.C., Meagher R.B., Dean J.F. (2005). Gene structure and molecular analysis of the laccase-like multicopper oxidase (LMCO) gene family in *Arabidopsis thaliana*. Planta.

[79] Stevens R., Truffault V., Baldet P., Gautier H. (2017). Ascorbate oxidase in plant growth, development, and stress tolerance. Ascorbic Acid in Plant Growth, Development Stress Tolerance.

[80] Yamamoto A., Bhuiyan M.N.H., Waditee R., Tanaka Y., Esaka M., Oba K., Jagendorf A.T., Takabe T. (2005). Suppressed expression of the apoplastic ascorbate oxidase gene increases salt tolerance in tobacco and *Arabidopsis* plants. J. Exp. Bot..

[81] Hoegger P.J., Kilaru S., James T.Y., Thacker J.R., Kües U. (2006). Phylogenetic comparison and classification of laccase and related multicopper oxidase protein sequences. FEBS J..

[82] Berthet S., Demont-Caulet N., Pollet B., Bidzinski P., Cézard L., Le Bris P., Borrega N., Hervé J., Blondet E., Balzergue S. (2011). Disruption of LACCASE4 and 17 results in tissue-specific alterations to lignification of *Arabidopsis thaliana* stems. Plant Cell.

[83] Hu Q., Xiao S., Guan Q., Tu L., Sheng F., Du X., Zhang X. (2020). The laccase gene *GhLac1* modulates fiber initiation and elongation by coordinating jasmonic acid and flavonoid metabolism. Crop J..

[84] Cai X., Davis E.J., Ballif J., Liang M., Bushman E., Haroldsen V., Torabinejad J., Wu Y. (2006). Mutant identification and characterization of the laccase gene family in *Arabidopsis*. J. Exp. Bot..

[85] Liang M., Haroldsen V., Cai X., Wu Y. (2006). Expression of a putative laccase gene, *ZmLAC1*, in maize primary roots under stress. Plant Cell Environ..

[86] Dong J., Kim S.T., Lord E.M. (2005). Plantacyanin plays a role in reproduction in *Arabidopsis*. Plant Physiol..

[87] Tyagi S., Shumayla, Singh S.P., Upadhyay S.K. (2019). Role of superoxide dismutases (SODs) in stress tolerance in plants. Molecular Approaches in Plant Biology Environmental Challenges.

[88] Liu C., Wang Y., Wang Y., Du Y., Song C., Song P., Yang Q., He F., Bai X., Huang L. (2022). Glycine-serine-rich effector *PstGSRE4* in *Puccinia striiformis* f. sp. tritici inhibits the activity of copper zinc superoxide dismutase to modulate immunity in wheat. PLoS Pathog..

[89] Huang C.-H., Kuo W.-Y., Weiss C., Jinn T.-L. (2012). Copper chaperone-dependent and-independent activation of three copper-zinc superoxide dismutase homologs localized in different cellular compartments in *Arabidopsis*. Plant Physiol..

[90] Chu C.-C., Lee W.-C., Guo W.-Y., Pan S.-M., Chen L.-J., Li H.-M., Jinn T.-L. (2005). A copper chaperone for superoxide dismutase that confers three types of copper/zinc superoxide dismutase activity in *Arabidopsis*. Plant Physiol..

[91] Valentine J.S., Gralla E.B. (1997). Delivering copper inside yeast and human cells. Science.

[92] Binder B.M. (2020). Ethylene signaling in plants. J. Biol. Chem..

[93] Huang J., Zhao X., Bürger M., Chory J., Wang X. (2023). The role of ethylene in plant temperature stress response. Trends Plant Sci..

[94] Husain T., Fatima A., Suhel M., Singh S., Sharma A., Prasad S.M., Singh V.P. (2020). A brief appraisal of ethylene signaling under abiotic stress in plants. Plant Signal. Behav..

[95] Azhar B.J., Abbas S., Aman S., Yamburenko M.V., Chen W., Müller L., Uzun B., Jewell D.A., Dong J., Shakeel S.N. (2023). Basis for high-affinity ethylene binding by the ethylene receptor ETR1 of *Arabidopsis*. Proc. Natl. Acad. Sci. USA.

[96] Kuper J., Llamas A., Hecht H.-J., Mendel R.R., Schwarz G. (2004). Structure of the molybdopterin-bound Cnx1G domain links molybdenum and copper metabolism. Nature.

[97] Hajiboland R. (2012). Effect of micronutrient deficiencies on plants stress responses. Abiotic Stress Responses in Plants: Metabolism, Productivity Sustainability.

[98] Marschner H. (2011). Chapter 7—Function of nutrients: Micronutrients A2—Marschner, Petra. Marschner’s Mineral Nutrition of Higher Plants.

[99] Yan J., Chia J.-C., Sheng H., Jung H.-I., Zavodna T.-O., Zhang L., Huang R., Jiao C., Craft E.J., Fei Z. (2017). *Arabidopsis* pollen fertility requires the transcription factors CITF1 and SPL7 that regulate copper delivery to anthers and jasmonic acid synthesis. Plant Cell.

[100] Batool I., Hassan I., Hasan S., Rafique T., Saleem A., Hussain T., Ali I. (2022). Qualitative and quantitative traits of sweet pepper as influenced by copper nanoparticles. Plant Cell Biotechnol. Mol. Biol..

[101] Eshghi S., Hashemi M., Mohammadi A., Badii F., Mohammadhoseini Z., Ahmadi K. (2014). Effect of nanochitosan-based coating with and without copper loaded on physicochemical and bioactive components of fresh strawberry fruit (*Fragaria x ananassa* Duchesne) during storage. Food Bioprocess Technol..

[102] Sharma B., Nigam S., Verma A., Garg M., Mittal A., Sadhu S.D. (2024). A Biogenic Approach to Develop Guava Derived Edible Copper and Zinc Oxide Nanocoating to Extend Shelf Life and Efficiency for Food Preservation. J. Polym. Environ..

[103] Majumdar S., Long R.W., Kirkwood J.S., Minakova A.S., Keller A.A. (2021). Unraveling metabolic and proteomic features in soybean plants in response to copper hydroxide nanowires compared to a commercial fertilizer. Environ. Sci. Technol..

[104] Yu Y., Liu H., Xia H., Chu Z. (2023). Double-or Triple-Tiered Protection: Prospects for the Sustainable Application of Copper-Based Antimicrobial Compounds for Another Fourteen Decades. Int. J. Mol. Sci..

[105] Printz B., Lutts S., Hausman J.-F., Sergeant K. (2016). Copper trafficking in plants and its implication on cell wall dynamics. Front. Plant Sci..

[106] Rehman M., Liu L., Wang Q., Saleem M.H., Bashir S., Ullah S., Peng D. (2019). Copper environmental toxicology, recent advances, and future outlook: A review. Environ. Sci. Pollut. Res..

[107] Fernández-Calviño D., Rodríguez-Suárez J.A., López-Periago E., Arias-Estévez M., Simal-Gándara J. (2008). Copper content of soils and river sediments in a winegrowing area, and its distribution among soil or sediment components. Geoderma.

[108] Brunetto G., de Melo G.W.B., Terzano R., Del Buono D., Astolfi S., Tomasi N., Pii Y., Mimmo T., Cesco S. (2016). Copper accumulation in vineyard soils: Rhizosphere processes and agronomic practices to limit its toxicity. Chemosphere.

[109] Lu Z., Zejiang C., Huiying W., Zikun Y., Tianfu H., Kailou L., Lisheng L., Jing H., Shilin W., Huimin Z. (2020). Distribution characteristics of effective medium and micronutrient element contents in paddy soils of China. Trans. Chin. Soc. Agric. Eng..

[110] Liu M., Lu S., Zeng Q. (2021). Research progress and development trends of cultivated land quality evaluation—Taking Qingyuan City’s cultivated land fertility as an example. Shanxi Agric. Econ..

[111] Zhang Z., Yang J., Song Y., He P. (2022). Evaluation on the soil quality and suitability of green food producing area in nehe city of heilongjiang province. Geol. Resour..

[112] Malhi S., Cowell L., Kutcher H. (2005). Relative effectiveness of various sources, methods, times and rates of copper fertilizers in improving grain yield of wheat on a Cu-deficient soil. Can. J. Plant Sci..

[113] Sinclair A.H., Edwards A.C. (2008). Micronutrient deficiency problems in agricultural crops in Europe. Micronutrient Deficiencies in Global Crop Production.

[114] Bradl H.B. (2004). Adsorption of heavy metal ions on soils and soils constituents. J. Colloid Interface Sci..

[115] Fernández M.A., Soulages O.E., Acebal S.G., Rueda E.H., Sánchez R.M.T. (2015). Sorption of Zn (II) and Cu (II) by four Argentinean soils as affected by pH, oxides, organic matter and clay content. Environ. Earth Sci..

[116] Ning Y., Zhang X., Li B., Wang Y., Guo J. (2019). Distribution of Cd and Cu fractions in Chinese soils and their relationships with soil pH: A meta-analysis. Sustainability.

[117] Vives-Peris V., De Ollas C., Gómez-Cadenas A., Pérez-Clemente R.M. (2020). Root exudates: From plant to rhizosphere and beyond. Plant Cell Rep..

[118] Ma W., Tang S., Dengzeng Z., Zhang D., Zhang T., Ma X. (2022). Root exudates contribute to belowground ecosystem hotspots: A review. Front. Microbiol..

[119] Lange B., van Der Ent A., Baker A.J.M., Echevarria G., Mahy G., Malaisse F., Meerts P., Pourret O., Verbruggen N., Faucon M.P. (2017). Copper and cobalt accumulation in plants: A critical assessment of the current state of knowledge. New Phytol..

[120] Ondrasek G., Begić H.B., Zovko M., Filipović L., Meriño-Gergichevich C., Savić R., Rengel Z. (2019). Biogeochemistry of soil organic matter in agroecosystems & environmental implications. Sci. Total Environ..

[121] Zamulina I.V., Gorovtsov A.V., Minkina T.M., Mandzhieva S.S., Burachevskaya M.V., Bauer T.V. (2022). Soil organic matter and biological activity under long-term contamination with copper. Environ. Geochem. Health.

[122] Barsova N., Yakimenko O., Tolpeshta I., Motuzova G. (2019). Current state and dynamics of heavy metal soil pollution in Russian Federation—A review. Environ. Pollut..

[123] Koegel-Knabner I., Rumpel C. (2018). Advances in molecular approaches for understanding soil organic matter composition, origin, and turnover: A historical overview. Adv. Agron..

[124] Araújo E., Strawn D.G., Morra M., Moore A., Alleoni L.R.F. (2019). Association between extracted copper and dissolved organic matter in dairy-manure amended soils. Environ. Pollut..

[125] Beesley L., Moreno-Jiménez E., Gomez-Eyles J.L. (2010). Effects of biochar and greenwaste compost amendments on mobility, bioavailability and toxicity of inorganic and organic contaminants in a multi-element polluted soil. Environ. Pollut..

[126] Li X., Zhang J., Gong Y., Liu Q., Yang S., Ma J., Zhao L., Hou H. (2020). Status of copper accumulation in agricultural soils across China (1985–2016). Chemosphere.

[127] Izydorczyk G., Mikula K., Skrzypczak D., Moustakas K., Witek-Krowiak A., Chojnacka K. (2021). Potential environmental pollution from copper metallurgy and methods of management. Environ. Res..

[128] Singh O., Labana S., Pandey G., Budhiraja R., Jain R. (2003). Phytoremediation: An overview of metallic ion decontamination from soil. Appl. Microbiol. Biotechnol..

[129] Wang M., Li S., Li X., Zhao Z., Chen S. (2018). An overview of current status of copper pollution in soil in China and remediation efforts research. Erath Sci. Front..

[130] Ortega P., Sanchez E., Gil E., Matamoros V. (2022). Use of cover crops in vineyards to prevent groundwater pollution by copper and organic fungicides. Soil column studies. Chemosphere.

[131] Lamichhane J.R., Osdaghi E., Behlau F., Köhl J., Jones J.B., Aubertot J.-N. (2018). Thirteen decades of antimicrobial copper compounds applied in agriculture. A review. Agron. Sustain. Dev..

[132] Yao T., Zhou Y., Zhou C. (2016). Advances in Copper Resistant Mechanisms and Control Methods of *Citrus* Canker. Acta Hortic. Sin..

[133] Triantafyllidis V., Zotos A., Kosma C., Kokkotos E. (2020). Environmental Implications from Long-term *Citrus* Cultivation and Wide Use of Cu Fungicides in Mediterranean Soils. Water Air Soil Pollut..

[134] Thomas G., Stärk H.-J., Wellenreuther G., Dickinson B.C., Küpper H. (2013). Effects of nanomolar copper on water plants—Comparison of biochemical and biophysical mechanisms of deficiency and sublethal toxicity under environmentally relevant conditions. Aquat. Toxicol..

[135] Yruela I. (2005). Copper in plants. Braz. J. Plant Physiol..

[136] Thomas G., Andresen E., Mattusch J., Hubáček T., Küpper H. (2016). Deficiency and toxicity of nanomolar copper in low irradiance—A physiological and metalloproteomic study in the aquatic plant *Ceratophyllum demersum*. Aquat. Toxicol..

[137] Cohu C.M., Pilon M. (2007). Regulation of superoxide dismutase expression by copper availability. Physiol. Plant.

[138] Yruela I. (2009). Copper in plants: Acquisition, transport and interactions. Funct. Plant Biol..

[139] Farid M., Farooq M.A., Fatima A., Abubakar M., Ali S., Raza N., Alhaithloul H.A.S., Soliman M.H. (2021). Copper-induced responses in different plant species. Approaches to the Remediation of Inorganic Pollutants.

[140] Ayala M.B., Gorgé J.L., Lachica M., Sandmann G. (1992). Changes in carotenoids and fatty acids in photosystem II of Cudeficient pea plants. Physiol. Plant.

[141] Lysenko E.A., Klaus A.A., Kartashov A.V., Kusnetsov V.V. (2020). Specificity of Cd, Cu, and Fe effects on barley growth, metal contents in leaves and chloroplasts, and activities of photosystem I and photosystem II. Plant Physiol. Biochem..

[142] Peng H., Kroneck P.M., Küpper H. (2013). Toxicity and deficiency of copper in *Elsholtzia splendens* affect photosynthesis biophysics, pigments and metal accumulation. Environ. Sci. Technol..

[143] Küpper H., Kroneck P.M. (2005). Heavy metal uptake by plants and cyanobacteria. Met. Ions Biol. Syst..

[144] Tian L., Li J., Huang C., Zhang D., Xu Y., Yang X., Song J., Wang D., Qiu N., Short D.P. (2021). Cu/Zn superoxide dismutase (VdSOD1) mediates reactive oxygen species detoxification and modulates virulence in *Verticillium dahliae*. Mol. Plant Pathol..

[145] Rahmati Ishka M., Vatamaniuk O.K. (2020). Copper deficiency alters shoot architecture and reduces fertility of both gynoecium and androecium in *Arabidopsis thaliana*. Plant Direct.

[146] Adrees M., Ali S., Rizwan M., Ibrahim M., Abbas F., Farid M., Zia-ur-Rehman M., Irshad M.K., Bharwana S.A. (2015). The effect of excess copper on growth and physiology of important food crops: A review. Environ. Sci. Pollut. Res..

[147] Ansari M.K.A., Oztetik E., Ahmad A., Umar S., Iqbal M., Owens G. (2013). Identification of the phytoremediation potential of Indian mustard genotypes for copper, evaluated from a hydroponic experiment. CLEAN-Soil Air Water.

[148] Wairich A., De Conti L., Lamb T.I., Keil R., Neves L.O., Brunetto G., Sperotto R.A., Ricachenevsky F.K. (2022). Throwing copper around: How plants control uptake, distribution, and accumulation of copper. Agronomy.

[149] Li L., Long M., Islam F., Farooq M.A., Wang J., Mwamba T.M., Shou J., Zhou W. (2019). Synergistic effects of chromium and copper on photosynthetic inhibition, subcellular distribution, and related gene expression in *Brassica napus* cultivars. Environ. Sci. Pollut. Res..

[150] Li Q., Chen H.-H., Qi Y.-P., Ye X., Yang L.-T., Huang Z.-R., Chen L.-S. (2019). Excess copper effects on growth, uptake of water and nutrients, carbohydrates, and PSII photochemistry revealed by OJIP transients in *Citrus* seedlings. Environ. Sci. Pollut. Res..

[151] Wodala B., Eitel G., Gyula T., Ördög A., Horváth F. (2012). Monitoring moderate Cu and Cd toxicity by chlorophyll fluorescence and P 700 absorbance in pea leaves. Photosynthetica.

[152] de Freitas T.A., França M.G.C., de Almeida A.-A.F., de Oliveira S.J.R., de Jesus R.M., Souza V.L., dos Santos Silva J.V., Mangabeira P.A. (2015). Morphology, ultrastructure and mineral uptake is affected by copper toxicity in young plants of *Inga subnuda* subs. luschnathiana (Benth.) TD Penn. Environ. Sci. Pollut. Res..

[153] Küpper H., Andresen E. (2016). Mechanisms of metal toxicity in plants. Metallomics.

[154] Husak V. (2015). Copper and copper-containing pesticides: Metabolism, toxicity and oxidative stress. J. Vasyl Stefanyk Precarpathian Natl. Univ..

[155] Bernal M., Roncel M., Ortega J.M., Picorel R., Yruela I. (2004). Copper effect on cytochrome b_559_ of photosystem II under photoinhibitory conditions. Physiol. Plant.

[156] Mohanty N., Vass I., Demeter S. (1989). Copper toxicity affects photosystem II electron transport at the secondary quinone acceptor, QB. Plant Physiol..

[157] Pätsikkä E., Kairavuo M., Šeršen F., Aro E.-M., Tyystjärvi E. (2002). Excess copper predisposes photosystem II to photoinhibition in vivo by outcompeting iron and causing decrease in leaf chlorophyll. Plant Physiol..

[158] Yruela I., Alfonso M., Barón M., Picorel R. (2000). Copper effect on the protein composition of photosystem II. Physiol. Plant.

[159] Anjum N.A., Sofo A., Scopa A., Roychoudhury A., Gill S.S., Iqbal M., Lukatkin A.S., Pereira E., Duarte A.C., Ahmad I. (2015). Lipids and proteins—Major targets of oxidative modifications in abiotic stressed plants. Environ. Sci. Pollut. Res..

[160] Gupta S.K., Sharma M., Deeba F., Pandey V. (2017). Role of reactive oxygen species in photophosphorylation and damage to D1 protein: Past and present. Reactive Oxygen Species in Plants.

[161] Rather B.A., Mir I.R., Masood A., Anjum N.A., Khan N.A. (2022). Ethylene-nitrogen synergism induces tolerance to copper stress by modulating antioxidant system and nitrogen metabolism and improves photosynthetic capacity in mustard. Environ. Sci. Pollut. Res..

[162] Al Mahmud J., Bhuyan M.B., Anee T.I., Nahar K., Fujita M., Hasanuzzaman M. (2019). Reactive oxygen species metabolism and antioxidant defense in plants under metal/metalloid stress. Plant Abiotic Stress Tolerance.

[163] Kapoor D., Singh S., Kumar V., Romero R., Prasad R., Singh J. (2019). Antioxidant enzymes regulation in plants in reference to reactive oxygen species (ROS) and reactive nitrogen species (RNS). Plant Gene.

[164] Quartacci M.F., Pinzino C., Sgherri C.L., Dalla Vecchia F., Navari-Izzo F. (2000). Growth in excess copper induces changes in the lipid composition and fluidity of PSII-enriched membranes in wheat. Physiol. Plant.

[165] Ali H., Assiri M.A., Shearn C.T., Fritz K.S. (2019). Lipid peroxidation derived reactive aldehydes in alcoholic liver disease. Curr. Opin. Toxicol..

[166] Yalcinkaya T., Uzilday B., Ozgur R., Turkan I., Mano J.i. (2019). Lipid peroxidation-derived reactive carbonyl species (RCS): Their interaction with ROS and cellular redox during environmental stresses. Environ. Exp. Bot..

[167] Upadhyay R., Panda S.K. (2010). Zinc reduces copper toxicity induced oxidative stress by promoting antioxidant defense in freshly grown aquatic duckweed *Spirodela polyrhiza* L.. J. Hazard. Mater..

[168] Ameh T., Sayes C.M. (2019). The potential exposure and hazards of copper nanoparticles: A review. Environ. Toxicol. Pharmacol..

[169] Feil S.B., Pii Y., Valentinuzzi F., Tiziani R., Mimmo T., Cesco S. (2020). Copper toxicity affects phosphorus uptake mechanisms at molecular and physiological levels in Cucumis sativus plants. Plant Physiol. Biochem..

[170] Roy S.K., Cho S.-W., Kwon S.J., Kamal A.H.M., Lee D.-G., Sarker K., Lee M.-S., Xin Z., Woo S.-H. (2017). Proteome characterization of copper stress responses in the roots of sorghum. Biometals.

[171] Rai S., Singh P.K., Mankotia S., Swain J., Satbhai S.B. (2021). Iron homeostasis in plants and its crosstalk with copper, zinc, and manganese. Plant Stress.

[172] Hippler F.W., Cipriano D.O., Boaretto R.M., Quaggio J.A., Gaziola S.A., Azevedo R.A., Mattos-Jr D. (2016). *Citrus* rootstocks regulate the nutritional status and antioxidant system of trees under copper stress. Environ. Exp. Bot..

[173] Yang Y., Fang X., Chen M., Wang L., Xia J., Wang Z., Fang J., Tran L.-S.P., Shangguan L. (2022). Copper stress in grapevine: Consequences, responses, and a novel mitigation strategy using 5-aminolevulinic acid. Environ. Pollut..

[174] Huo K., Shangguan X., Xia Y., Shen Z., Chen C. (2020). Excess copper inhibits the growth of rice seedlings by decreasing uptake of nitrate. Ecotoxicol. Environ. Saf..

[175] Xin X., Zhao F., Rho J.Y., Goodrich S.L., Sumerlin B.S., He Z. (2020). Use of polymeric nanoparticles to improve seed germination and plant growth under copper stress. Sci. Total Environ..

[176] Ahsan N., Lee D.-G., Lee S.-H., Kang K.Y., Lee J.J., Kim P.J., Yoon H.-S., Kim J.-S., Lee B.-H. (2007). Excess copper induced physiological and proteomic changes in germinating rice seeds. Chemosphere.

[177] Kul R., Ekinci M., Turan M., Ors S., Yildirim E. (2021). How abiotic stress conditions affects plant roots. Plant Roots.

[178] Sanchez-Viveros G., Gonzalez-Mendoza D., Alarcon A., Ferrera-Cerrato R. (2010). Copper effects on photosynthetic activity and membrane leakage of *Azolla filiculoides* and *A. caroliniana*. Int. J. Agric. Biol..

[179] Marques D., Da Silva A., Mantovani J., Magalhães P., De Souza T. (2019). Root morphology and leaf gas exchange in *Peltophorum dubium* (Spreng.) Taub.(Caesalpinioideae) exposed to copper-induced toxicity. S. Afr. J. Bot..

[180] Lequeux H., Hermans C., Lutts S., Verbruggen N. (2010). Response to copper excess in *Arabidopsis thaliana*: Impact on the root system architecture, hormone distribution, lignin accumulation and mineral profile. Plant Physiol. Biochem..

[181] Lu F., Hu P., Lin M., Ye X., Chen L., Huang Z. (2022). Photosynthetic characteristics and chloroplast ultrastructure responses of citrus leaves to copper toxicity induced by Bordeaux mixture in greenhouse. Int. J. Mol. Sci..

[182] Tahjib-Ul-Arif M., Sohag A.A.M., Mostofa M., Polash M., Mahamud A., Afrin S., Hossain M., Hossain M., Murata Y., Tran L.S. (2021). Comparative effects of ascobin and glutathione on copper homeostasis and oxidative stress metabolism in mitigation of copper toxicity in rice. Plant Biol..

[183] Bouazizi H., Jouili H., Geitmann A., El Ferjani E. (2010). Copper toxicity in expanding leaves of *Phaseolus vulgaris* L.: Antioxidant enzyme response and nutrient element uptake. Ecotoxicol. Environ. Saf..

[184] Saleem M.H., Ali S., Irshad S., Hussaan M., Rizwan M., Rana M.S., Hashem A., Abd_Allah E.F., Ahmad P. (2020). Copper uptake and accumulation, ultra-structural alteration, and bast fibre yield and quality of fibrous jute (*Corchorus capsularis* L.) plants grown under two different soils of China. Plants.

[185] Ishka M.R., Chia J.-C., Vatamaniuk O.K., Upadhyay S.K. (2022). Chapter 12—Advances in understanding of copper function and transport in plants. Cation Transporters in Plants.

[186] Meychik N., Nikolaeva Y., Kushunina M. (2021). The significance of ion-exchange properties of plant root cell walls for nutrient and water uptake by plants. Plant Physiol. Biochem..

[187] Zhang B., Gao Y., Zhang L., Zhou Y. (2021). The plant cell wall: Biosynthesis, construction, and functions. J. Integr. Plant Biol..

[188] Sasaki A., Yamaji N., Ma J.F. (2016). Transporters involved in mineral nutrient uptake in rice. J. Exp. Bot..

[189] Yamaji N., Ma J.F. (2014). The node, a hub for mineral nutrient distribution in graminaceous plants. Trends Plant Sci..

[190] Chen G., Li J., Han H., Du R., Wang X. (2022). Physiological and Molecular Mechanisms of Plant Responses to Copper Stress. Int. J. Mol. Sci..

[191] Pilon M., Cohu C.M., Ravet K., Abdel-Ghany S.E., Gaymard F. (2009). Essential transition metal homeostasis in plants. Curr. Opin. Plant Biol..

[192] Kumar V., Pandita S., Singh Sidhu G.P., Sharma A., Khanna K., Kaur P., Bali A.S., Setia R. (2021). Copper bioavailability, uptake, toxicity and tolerance in plants: A comprehensive review. Chemosphere.

[193] De Feo C.J., Aller S.G., Siluvai G.S., Blackburn N.J., Unger V.M. (2009). Three-dimensional structure of the human copper transporter hCTR1. Proc. Natl. Acad. Sci. USA.

[194] Sancenón V., Puig S., Mira H., Thiele D.J., Peñarrubia L. (2003). Identification of a copper transporter family in *Arabidopsis thaliana*. Plant Mol. Biol..

[195] Williams L.E., Mills R.F. (2005). P_1B_-ATPases--an ancient family of transition metal pumps with diverse functions in plants. Trends Plant Sci..

[196] Guerinot M.L. (2000). The ZIP family of metal transporters. Biochim. Biophys. Acta Bioenerg..

[197] Conte S.S., Walker E.L. (2012). Genetic and biochemical approaches for studying the yellow stripe-like transporter family in plants. Curr. Top. Membr..

[198] O’Halloran T.V., Culotta V.C. (2000). Metallochaperones, an intracellular shuttle service for metal ions. J. Biol. Chem..

[199] Zhang Y., Chen K., Zhao F.-J., Sun C., Jin C., Shi Y., Sun Y., Li Y., Yang M., Jing X. (2018). OsATX1 interacts with heavy metal P_1B_-type ATPases and affects copper transport and distribution. Plant Physiol..

[200] Satbhai S.B., Setzer C., Freynschlag F., Slovak R., Kerdaffrec E., Busch W. (2017). Natural allelic variation of FRO2 modulates *Arabidopsis* root growth under iron deficiency. Nat. Commun..

[201] Rodrigues W.F.C., Lisboa A.B.P., Lima J.E., Ricachenevsky F.K., Del-Bem L.E. (2023). Ferrous iron uptake via IRT1/ZIP evolved at least twice in green plants. New Phytol..

[202] Bernal M., Casero D., Singh V., Wilson G.T., Grande A., Yang H., Dodani S.C., Pellegrini M., Huijser P., Connolly E.L. (2012). Transcriptome sequencing identifies SPL7-regulated copper acquisition genes FRO4/FRO5 and the copper dependence of iron homeostasis in *Arabidopsis*. Plant Cell.

[203] Sancenón V., Puig S., Mateu-Andrés I., Dorcey E., Thiele D.J., Peñarrubia L. (2004). The *Arabidopsis* copper transporter COPT1 functions in root elongation and pollen development. J. Biol. Chem..

[204] Andrés-Colás N., Perea-García A., Puig S., Peñarrubia L. (2010). Deregulated copper transport affects *Arabidopsis* development especially in the absence of environmental cycles. Plant Physiol..

[205] Flockerzi V., Beck A., Offermanns S., Rosenthal W. (2020). Non-selective Cation Channels. Encyclopedia of Molecular Pharmacology.

[206] Yamasaki H., Hayashi M., Fukazawa M., Kobayashi Y., Shikanai T. (2009). SQUAMOSA Promoter Binding Protein-Like7 Is a Central Regulator for Copper Homeostasis in *Arabidopsis*. Plant Cell.

[207] Yuan M., Chu Z., Li X., Xu C., Wang S. (2010). The bacterial pathogen *Xanthomonas oryzae* overcomes rice defenses by regulating host copper redistribution. Plant Cell.

[208] Yuan M., Li X., Xiao J., Wang S. (2011). Molecular and functional analyses of COPT/Ctr-type copper transporter-like gene family in rice. BMC Plant Biol..

[209] Ando Y., Nagata S., Yanagisawa S., Yoneyama T. (2012). Copper in xylem and phloem saps from rice (*Oryza sativa*): The effect of moderate copper concentrations in the growth medium on the accumulation of five essential metals and a speciation analysis of copper-containing compounds. Funct. Plant Biol..

[210] Chu H.H., Chiecko J., Punshon T., Lanzirotti A., Lahner B., Salt D.E., Walker E.L. (2010). Successful reproduction requires the function of *Arabidopsis* Yellow Stripe-Like1 and Yellow Stripe-Like3 metal-nicotianamine transporters in both vegetative and reproductive structures. Plant Physiol..

[211] Zheng L., Yamaji N., Yokosho K., Ma J.F. (2012). YSL16 is a phloem-localized transporter of the copper-nicotianamine complex that is responsible for copper distribution in rice. Plant Cell.

[212] Guan M., Zhang W., Xu P., Zhao Q., Chen M., Cao Z. (2022). Mapping and functional analysis of high-copper accumulation mutant *oshc1* in rice. J. Hazard. Mater..

[213] Huang X.Y., Deng F., Yamaji N., Pinson S.R., Fujii-Kashino M., Danku J., Douglas A., Guerinot M.L., Salt D.E., Ma J.F. (2016). A heavy metal P-type ATPase OsHMA4 prevents copper accumulation in rice grain. Nat. Commun..

[214] Migocka M., Posyniak E., Maciaszczyk-Dziubinska E., Papierniak A., Kosieradzaka A. (2015). Functional and Biochemical Characterization of Cucumber Genes Encoding Two Copper ATPases CsHMA5.1 and CsHMA5.2. J. Biol. Chem..

[215] He C., Berkowitz O., Hu S., Zhao Y., Qian K., Shou H., Whelan J., Wang Y. (2023). Co-regulation of mitochondrial and chloroplast function: Molecular components and mechanisms. Plant Commun..

[216] Blaby-Haas C.E., Padilla-Benavides T., Stübe R., Argüello J.M., Merchant S.S. (2014). Evolution of a plant-specific copper chaperone family for chloroplast copper homeostasis. Proc. Natl. Acad. Sci. USA.

[217] Horng Y.C., Cobine P.A., Maxfield A.B., Carr H.S., Winge D.R. (2004). Specific copper transfer from the Cox17 metallochaperone to both Sco1 and Cox11 in the assembly of yeast cytochrome C oxidase. J. Biol. Chem..

[218] Steinebrunner I., Landschreiber M., Krause-Buchholz U., Teichmann J., Rödel G. (2011). HCC1, the *Arabidopsis* homologue of the yeast mitochondrial copper chaperone SCO1, is essential for embryonic development. J. Exp. Bot..

[219] Steinebrunner I., Gey U., Andres M., Garcia L., Gonzalez D.H. (2014). Divergent functions of the *Arabidopsis* mitochondrial SCO proteins: HCC1 is essential for COX activity while HCC2 is involved in the UV-B stress response. Front. Plant Sci..

[220] Radin I., Mansilla N., Rödel G., Steinebrunner I. (2015). The *Arabidopsis* COX11 homolog is essential for cytochrome c oxidase activity. Front. Plant Sci..

[221] Binder B.M., Rodríguez F.I., Bleecker A.B. (2010). The copper transporter RAN1 is essential for biogenesis of ethylene receptors in *Arabidopsis*. J. Biol. Chem..

[222] Rodríguez F.I., Esch J.J., Hall A.E., Binder B.M., Schaller G.E., Bleecker A.B. (1999). A copper cofactor for the ethylene receptor ETR1 from *Arabidopsis*. Science.

[223] Woeste K.E., Kieber J.J. (2000). A strong loss-of-function mutation in *RAN1* results in constitutive activation of the ethylene response pathway as well as a rosette-lethal phenotype. Plant Cell.

[224] Hu X., Wang S., Zhang H., Zhang H., Feng S., Qiao K., Lv F., Gong S., Zhou A. (2022). Plant cadmium resistance 6 from *Salix linearistipularis* (SlPCR6) affects cadmium and copper uptake in roots of transgenic Populus. Ecotoxicol. Environ. Saf..

[225] Schulten A., Pietzenuk B., Quintana J., Scholle M., Feil R., Krause M., Romera-Branchat M., Wahl V., Severing E., Coupland G. (2022). Energy status-promoted growth and development of *Arabidopsis* require copper deficiency response transcriptional regulator SPL7. Plant Cell.

[226] Garcia-Molina A., Xing S., Huijser P. (2014). Functional characterisation of *Arabidopsis* SPL7 conserved protein domains suggests novel regulatory mechanisms in the Cu deficiency response. BMC Plant Biol..

[227] Garcia-Molina A., Xing S., Huijser P. (2014). A conserved KIN17 curved DNA-binding domain protein assembles with SQUAMOSA PROMOTER-BINDING PROTEIN-LIKE7 to adapt *Arabidopsis* growth and development to limiting copper availability. Plant Physiol..

[228] Zhang H., Zhao X., Li J., Cai H., Deng X.W., Li L. (2014). *MicroRNA408* is critical for the *HY5-SPL7* gene network that mediates the coordinated response to light and copper. Plant Cell.

[229] Abdel-Ghany S.E., Pilon M. (2008). *MicroRNA*-mediated systemic down-regulation of copper protein expression in response to low copper availability in *Arabidopsis*. J. Biol. Chem..

[230] Perea-García A., Andrés-Bordería A., Mayo de Andres S., Sanz A., Davis A.M., Davis S.J., Huijser P., Peñarrubia L. (2016). Modulation of copper deficiency responses by diurnal and circadian rhythms in *Arabidopsis thaliana*. J. Exp. Bot..

[231] Andrés-Colás N., Carrió-Seguí A., Abdel-Ghany S.E., Pilon M., Peñarrubia L. (2018). Expression of the Intracellular COPT3-Mediated Cu Transport Is Temporally Regulated by the TCP16 Transcription Factor. Front. Plant Sci..

[232] Ji C., Li H., Ding J., Yu L., Jiang C., Wang C., Wang S., Ding G., Shi L., Xu F. (2024). Rice transcription factor OsWRKY37 positively regulates flowering time and grain fertility under copper deficiency. Plant Physiol..

[233] Cai Y., Li Y., Liang G. (2021). FIT and bHLH Ib transcription factors modulate iron and copper crosstalk in *Arabidopsis*. Plant Cell Environ..

[234] Hao C., Yang Y., Du J., Deng X.W., Li L. (2022). The PCY-SAG14 phytocyanin module regulated by PIFs and miR408 promotes dark-induced leaf senescence in *Arabidopsis*. Proc. Natl. Acad. Sci. USA.

